# Frailty or resilience? Hazard-based and cumulative phenotype approaches to discerning signals of health inequality in medieval London

**DOI:** 10.1126/sciadv.adq5703

**Published:** 2024-11-13

**Authors:** Samantha L. Yaussy, Kathryn E. Marklein, Sharon N. DeWitte, Douglas E. Crews

**Affiliations:** ^1^ Department of Sociology and Anthropology, James Madison University, Harrisonburg, VA, USA.; ^2^ Department of Anthropology, University of Louisville, Louisville, KY, USA.; ^3^ Center for Archaeology and Cultural Heritage, University of Louisville, Louisville, KY, USA.; ^4^ Department of Anthropology, University of Colorado Boulder, Boulder, CO, USA.; ^5^ Institute of Behavioral Science, University of Colorado Boulder, Boulder, CO, USA.; ^6^ Department of Anthropology, The Ohio State University, Columbus, OH, USA.; ^7^ College of Public Health, The Ohio State University, Columbus, OH, USA.

## Abstract

Bioarchaeology uses human skeletal remains to reconstruct varied experiences of individuals and populations in the past, including patterns of health across time periods and cultural contexts. In the past three decades, bioarchaeological studies have highlighted the concept of “frailty,” operationalizing it as increased risk of mortality or cumulative phenotypes. Using data from medieval London cemeteries, we integrate these two approaches to frailty in past populations. First, we estimate the risks of mortality and survivorship (hazard and survival analyses) associated with 10 biomarkers and use these results to construct population-specific frailty and resilience indices. Then, we apply the indices to adult individuals to explore frailty and resilience differentials between males and females in medieval London. Findings suggest that the male-female morbidity-mortality paradox observed in modern populations may not have existed in this context, which may be explained by preferential cultural buffering of men in this patriarchal, urban setting.

## Teaser

Integrating approaches to studying frailty in the past generates separate frailty and resilience indices for medieval London.

## INTRODUCTION

Derived from the Latin term for fragile (*fragilis*), frail entered the Middle English (1100 to 1500 CE) canon to describe dependent elders and individuals showing physical and moral weakness and continuing poor health. Today, frailty is a common concept peppered throughout everyday speech. In modern health care settings, frailty is a diagnosable phenotype of reduced strength, endurance, and mobility, increased susceptibility to fatigue, and decreased resilience to disease, illness, and injury [see (*1*)]. During life, exposures to extrinsic (e.g., occupational, accidental, environmental, and sociocultural), intrinsic (e.g., DNA/genes, cell biology, and somatic fragility), and ascribed and sociocultural stressors (e.g., sex, gender, income, occupation, social status, and residential setting) promote frailty across bodily systems (e.g., muscular, neurological, cardiovascular, and skeletal). Whether observed visually or assessed scalarly, physical frailty accumulates over the later decades of human life. Because Fried and colleagues (*1*) developed the original physical frailty index, frailty as a concept along with multiple additional frailty indices have been applied across medicine and eldercare for monitoring patients’ abilities, need for health care, and accommodations (*2*, *3*). Still, neither “frailty” nor “frailty index” were included as terms in two recent compendiums of medical terminology (*4*, *5*) nor is there a consensus definition of frailty or its measurement among the living or dead.

Unlike the long historical and common use of frailty in medical care settings, assessments of frailty in human bioarchaeological research have a relatively recent history. Frailty, as a concept (not condition), was spotlighted in Wood and colleagues’ (*6*) paradigmatic article, which introduced the osteological paradox. Since this paper, frailty in human bioarchaeology has been assessed within and compared across multiple groups of people using visual and metric assessments and implemented using differing conceptualizations of and methods for quantification. Generally, in bioarchaeology, frailty has been defined in accordance with demographic usage (*7*) and operationalized as increased risk of mortality (*8*–*11*); recent reconceptualizations of frailty in bioarchaeology consider frailty as a cumulative phenotype (*12*–*14*). These differing (but potentially complementary) approaches nonetheless have demonstrated aspects of the osteological paradox, i.e., how skeletal markers of stressors occurring during life may be indicators of frailty or resilience within and across populations. These approaches provide avenues toward better engaging with the osteological paradox. Given recent developments in assessing frailty in both the living and the long deceased, now is an appropriate time to review research articulating frailty in bioarchaeology and human biology, interrogate frailty as mortality risk and a phenotype within the osteological paradox, and outline research methodologies for assessing skeletal frailty across and between populations.

Here, we first explore how and why modern health care specialists and bioarchaeologists seek to understand and assess frailty among the living and dead, respectively, and where overlaps in these areas exist for productive interdisciplinary dialogue. By exploring how each discipline, using varying methods and samples, has developed research methods and modeling techniques to assess physical or physiological frailty, we show how they jointly inform research methods and understanding of the consequences of life’s stressors across time and space. To do so, we review variation in how frailty is defined in the living by assessing whole-body factors and among skeletal remains via the presence, absence, and nature (e.g., severity) of pathological conditions. We also explore how skeletal frailty may be useful in exploring associations between health and differentials in burial sites, types of burial, and the presence of grave goods and the usefulness of assessing skeletal frailty within and across groups of individuals. Last, we explore and test current and emerging methods for estimating variability in levels of frailty within and among skeletal assemblages, including analysis of mortality and survivorship, and cumulative (e.g., frailty index) methods (*8*, *9*, *11*–*13*, *15*–*17*). Following this review, we apply these analytical methods in an exploratory analysis to assess skeletal frailty differentials between estimated females and males from medieval London and to demonstrate the expansive and nuanced interpretive potential of combining these approaches in bioarchaeological research.

## BACKGROUND

### Frailty in the living

Within geriatric medicine and gerontology, physical frailty is an observable phenotype of declining strength, endurance, physical activity, body weight, and physical abilities occurring during late life (*1*, *18*). The physical frailty phenotype reflects progressive atrophy of muscle (sarcopenia) and bone loss (osteopenia and osteoporosis) leading to declines in physical abilities and body size during our later decades of life (*1*, *3*, *18*–*20*). To support health care assessment, delivery, and research among seniors, multiple frailty indices have been developed [see (*21*)]. Commonly, these include similar directly accessible quantitative assessments of physical/functional traits. However, they may also include physical capacities and limitations, along with cognitive, mental, and health assessments (*20*, *22*, *23*) and be scored as reductions/deficits in capacity or function. Most traits for estimating frailty in the living are not directly assessable among the long deceased. Still, stressors and processes leading to age-related increases in frailty today also likely affected past people. Although proceeding at variable rates between individuals, physical frailty increases as survival extends into our later decades as it likely did among our ancestors.

Fried and colleagues (*1*) described physical frailty as a clinical phenotype, developing a frailty index including five assessments: low muscle strength, endurance, and physical activity, slow walking speed, and recent unintended weight loss. Since their publication, frailty indices including different and additional assessments and indexing methods have been reported (*3*, *21*, *23*, *24*). Many factors contribute to the muscle atrophy, bone loss, limited strength, physical inactivity, and mobility defined as frailty, making it a predictable, measurable, and assessable phenotype (*25*, *26*). Although reporting no consensus definition, a Delphi method–based consensus statement (*27*) observed that the most frequently reported frailty model is the five-domain physical frailty index (*1*). On the basis of the five-factor physical frailty index, patients exhibiting three or more criteria were identified as frail, others not frail (*1*). Most subsequent reports followed this methodology, some adding functional limitations such as difficulties walking, climbing steps, or carrying objects, others adding assessments like hospital admissions, comorbidities, self-reported mobility and health issues, cognitive function, mental health, and accumulated physical and mental deficits (*23*, *25*, *28*, *29*).

As a multidimensional phenotype reflecting reduced energy availability/expenditure, decreased physical abilities, cognition, health, and reserve capacity, frailty is observable at multiple levels (*1*, *18*, *23*). Within clinical and congregate care settings, frailty indices include an array of assessments beyond the original five factors proposed by Fried and colleagues (*18*). Rockwood and colleagues (*23*) developed a Clinical Frailty Scale, based on a battery of health assessments [e.g., a 70-item clinical deficits frailty index, a modified Mini-Mental State Exam, a cognitive scale, a function scale, and cognitive impairment questions from the Diagnostic and Statistical Manual of Mental Disorders, 3rd Edition (Revised)], that ranges from “robust health” to “complete functional dependency on others.” Similarly, Studenski and colleagues (*24*) developed the Clinical Global Impression of Change in Physical Frailty based on clinical judgment including six intrinsic domains (mobility, balance, strength, endurance, nutrition, and neuromotor performance) and seven consequences domains (medical complexity, health care utilization, appearance, self-perceived health, activities of daily living, emotional status, and social status). Both Rockwood and colleagues’ (*23*) and Studenski and colleagues’ (*24*) frailty assessments are deficit scales based on assessing individual inabilities to perform, achieve, or respond to a series of physical, cognitive, functional, and physiological assessments.

As an outcome of lifelong physical changes secondary to experienced stressors, frailty increases with age, most rapidly after our sixth decade, achieving its highest frequency at ages 70+ years. However, physical frailty is also observed at younger ages. For example, in a recent cross-sectional national cohort study in India, based on a 40-item deficit frailty index, frailty was present among 30% of men and women aged 45+ years (*30*). Across multiple cohort studies, among those aged 70+ years, frailty associates significantly with physiological dysregulation (*18*, *28*, *29*, *31*). In this way, frailty may be viewed as later-life outcomes/markers of resilience and survival following lifelong exposures to stressors and increasing risks for morbidity and mortality others have not survived (*32*). As a state of increasing vulnerability emerging independent of any specific disease or illness (*18*), estimating frailty among the deceased based on skeletal indicators of stress or embodied experiences provides a window for exploring age, status, sex-based, and gendered risks in the past. In the living, physical frailty reflects a constellation of stressors occurring throughout life leading to physiological dysregulation, reduced physical abilities, declining reserve capacity, and reduced resilience with increasing age (*1*).

### Osteological paradox

While understanding and mitigating frailty in the living is critical to clinical applications and medical anthropology, physical frailty indices and measures of frailty are not directly applicable to the dead. Bioarchaeological research on health and disease in the past must confront several fundamental limitations associated with data derived from human skeletal remains. Some of those limitations are encompassed within the osteological paradox, which, as detailed by Wood and colleagues (*6*), refers to the inherent uncertainty we encounter when trying to reconstruct individual or population-level patterns of health or variation thereof from biased skeletal samples. This complicates inferences about individual or subgroup-level susceptibility to disease and the experiences and consequences of diseases and other disorders in the past, particularly when we use the presence and absence of skeletal lesions as measures of health. As invaluable as human skeletal remains are for reconstructing life in the past, they are far from perfect reflections of the once-living people or populations we seek to understand, and some of that imperfection derives from the effects of heterogeneous frailty and selective mortality. Heterogeneous frailty refers to intrapopulation variation in frailty, which was originally defined by Vaupel and colleagues (*7*) as an individual’s age-standardized risk of dying compared to others in the population but has been used more generally to refer to susceptibility to disease and death (*6*). Frailty has been also defined in bioarchaeology in ways more explicitly aligned with models of frailty used in modern clinical settings (see section Frailty as skeletal phenotype). Reconstructions of health in the past would be much simpler, if perhaps a bit boring, if everyone within a particular population experienced the same frailty. However, it is clear from observations of living people and from historical data that people vary in frailty, and presumably this was also the case in the more distant past. This heterogeneous frailty is the result of variation in the myriad factors that give rise to risks of death and disease, such as nutritional status, immunocompetence, genetics and epigenetics, hormones, propensity to engage in risk-taking behavior, occupational hazards, socioeconomic status, exposure to disease vectors, or environmental pollution. Selective mortality refers to the fact that most causes of death are selective with respect to frailty, i.e., the individuals who die at each age are disproportionately those with the highest frailty at that age. Individuals with lower frailty at each age are more likely to survive that particular age and die at later ages. Thus, individuals who comprise the skeletal samples studied by bioarchaeologists might (at best) represent the frailest segments of past societies and not health conditions in the living population in general.

Wood and colleagues (*6*) emphasized that much of the heterogeneous frailty that exists in living populations is unobservable in human skeletal remains. Although some aspects of our biosocial identities and experiences might be inferred from burial context or are embodied, leaving biochemical traces or producing physical changes on and in our skeletons, there are others that leave no such evidence. Because of this “hidden” heterogeneity in frailty and the selective mortality that occurs as a result, the aggregate patterns observed in skeletal samples can mask a great deal of underlying heterogeneity and might theoretically be compatible with multiple, equally plausible, perhaps even diametrically opposed, scenarios regarding patterns of health in the living population at the individual and subpopulation levels. Wood and colleagues (*6*) discuss the possibility that, because skeletal pathologies take time to form, they reflect survival (at least over the short term) of the causative etiologies. Thus, skeletal pathologies might be viewed as measures of resilience and, in at least some cases, indicate relatively good health rather than high frailty as they are more conventionally viewed. Conversely, individuals who lack skeletal pathological lesions might have been in poor health and thus succumbed to causative stressors before lesions formed. Wood and colleagues (*6*), therefore, warned against premature conclusions that skeletal lesions necessarily indicate poor (or good) health and encouraged clarification of the sources of frailty and the shapes and consequences of frailty distributions. These concerns should not discourage us from attempting to study health and disease in the past using skeletal data, but they should motivate us to be cautious in our interpretations and to use as many lines of evidence as possible.

Another challenge we face in studies of health in the past is the relatively low (or, in many cases, undetermined) sensitivity and specificity of skeletal pathological indicators. With respect to skeletal pathologies and disease, sensitivity is the proportion of people with a disease who are correctly identified as having it based on the presence of a particular skeletal lesion, and specificity is the proportion of people without a disease who are correctly identified as not having it based on the absence of a particular skeletal lesion (*33*, *34*); i.e., specificity is the extent to which people who have a skeletal pathology really represent the disease of interest. Bioarchaeologists rely upon skeletal pathological indicators, or stress markers, for which there is often limited, if any, information about sensitivity and specificity. The cellular, chemical, and biomechanical properties of bone mean that it responds to disease and other stressors in a limited number of ways. In general, bone may be deposited, removed, or deformed, and although there can be variation in the amount, severity, and distribution of these bony changes across etiologies, there are fundamental limits to the specificity of skeletal pathological conditions. As a result, several diseases can cause skeletal lesions that look similar, if not identical [see, e.g., (*35*)]. For those that produce pathognomonic lesions, differential diagnosis may not be possible if skeletal remains are incomplete or poorly preserved. The sensitivity of skeletal lesions is limited because not everyone with conditions that have the potential to affect the skeleton actually produces skeletal lesions (*33*). The few paleoepidemiological studies that have estimated the sensitivity and specificity of skeletal lesions include those by Smith-Guzmán (*36*) and Dangvard Pedersen and colleagues (*37*). For example, Smith-Guzmán (*36*) assessed the sensitivity and specificity of a suite of skeletal lesions with respect to malaria-associated anemia using clinical samples of individuals with known cause of death or malaria exposure. With the exception of spinal porosity, which had roughly comparable sensitivity and specificity (0.894 and 0.816, respectively), four of the five other indicators they tested (cribra orbitalia, humeral cribra, femoral cribra, and periostitis) had much higher specificity than sensitivity, indicating that these biomarkers produce relatively high rates of false positives but relatively low rates of false negatives. Porotic hyperostosis, conversely, had lower specificity than sensitivity (*36*).

In light of these issues, Wood and colleagues (*6*) call for work that clarifies the mechanisms underlying skeletal indicators of stress and disease. To make accurate assessments of health in the past, we need to know much more about how the formation and expression of stress markers are affected by underlying pathological processes and host characteristics (including immune function and history of stress exposures) and how the mechanisms that produce skeletal lesions affect later-life outcomes. Work along these lines is emerging. For example, O’Donnell and colleagues (*38*), using autopsy and medical history data from the New Mexico Office of the Medical Investigator, assess the association between porous cranial lesions (PCLs; e.g., cribra orbitalia and porotic hyperostosis) and length of childhood illness. They find, among other things, that individuals sick for over a month are more likely to have PCLs and that PCL morphology varies depending on the length and general category of illness. Crespo and colleagues’ (*39*) osteoimmunological work also addresses this general topic, focusing on how infection with one pathogen affects systemic inflammation and susceptibility to and immune and osteological responses to subsequent pathogen exposure.

Informative work engaging with the osteological paradox is possible, even in the face of incomplete understanding of the complexities of lesion formation processes. We focus here on demographic approaches to detecting heterogeneity in frailty and selective mortality using survival or hazards analysis, as detailed below.

### Frailty as risk of mortality

Although of interest to bioarchaeologists and other scholars, frailty is not defined or conceptualized in similar manners across or even within disciplines. In demographic scholarship, frailty was originally defined by Vaupel and colleagues (*7*) as an individual’s relative risk of death compared to other members of the population. In other words, frailty refers to the collection of susceptibilities and risk factors that alters an individual’s risks of death at different ages (*40*). As a result, it is impossible to divorce the biodemographic concept of frailty from the risks of mortality that exist in a given context. However, as described above, the differential susceptibility to disease and death that exists among individuals in a population (i.e., their frailty) will be influenced by a variety of genetic, biological, environmental, and cultural factors, all of which vary across space and time (*6*). As previously discussed, both the observable and hidden aspects of heterogeneity in frailty will affect patterns of mortality in a population and, if not controlled for statistically, can make it difficult to assess and interpret patterns of health or risk among individuals or subpopulations contained within the larger population or sample.

Much of the bioarchaeological research conducted on frailty has been designed around the tasks set forth by Wood and colleagues (*6*) in their paper on the challenges of the osteological paradox. First, the authors suggest that research be conducted with modern populations to identify the sources and distributions of frailty among living people and establish how a given distribution of frailty relates to the distribution of risks of death among individuals—although Wood and colleagues (*6*) recognize that these tasks may be beyond the purview of the field of bioarchaeology per se. Regardless, some bioarchaeologists have undertaken this task, using contemporary skeletal collections with known medical histories to investigate how skeletal lesions relate to susceptibility to particular diseases or risk of death [e.g., (*41*, *42*)]. Wood and colleagues (*6*) further suggest that bioarchaeologists can most meaningfully contribute to an understanding of how cultural context influences heterogeneity in frailty and, by extension, selective mortality.

In this vein, two complementary approaches have been developed to investigate the association between frailty and mortality using data gathered from human skeletal samples. One approach to studying frailty and mortality is the use of age-structured data to study selective mortality, which is ultimately driven by heterogeneous frailty. These studies primarily use hazards analysis or survival analysis to evaluate associations among skeletal stress markers and risks of mortality or survivorship [e.g., (*8*, *9*, *43*–*45*)]. In this approach, skeletal stress markers, or other factors, associated with higher hazards of death or lower survivorship are viewed as reflecting high frailty or poor health. Those associated with lower hazards of death or higher survivorship reflect low frailty or good health. Examples of this approach include work by DeWitte and colleagues (*9*, *46*), Kelmelis and colleagues (*47*), Yaussy and colleagues (*11*), Ham and colleagues (*48*), Wyatt and colleagues (*16*), and Wissler and DeWitte (*49*). For example, Godde, Pasillas, and Sanchez (*10*) use Cox proportional hazards regression to estimate the hazards ratios of dying of the Black Death in medieval London, and their results indicated a 1.67-fold increase in hazards for individuals with one or more skeletal indicators of frailty compared to those without such lesions. Other studies have considered the effects of variables beyond skeletal lesions—including sex, socioeconomic status, and archaeological proxies for identity or social position—to inform our understanding of frailty and selective mortality. For instance, DeWitte and colleagues (*50*) use the Siler model of mortality and Kaplan-Meier survival analysis to evaluate if socioeconomic status affected the risks of mortality and survivorship in industrializing London, and their results indicate increased mortality and reduced survivorship among children of low socioeconomic status. The study by DeWitte and colleagues (*50*) emphasizes how social patterns potentially affect patterns of heterogeneous frailty and selective mortality in past populations, reiterating the critical consideration of cultural context in such bioarchaeological studies.

The second approach to studying frailty as risk of mortality examines the relationship between survival and lesion activity to better understand how the stages of skeletal lesion formation and healing are associated with frailty and resilience. Scholars have called for a focus on the variations in frailty that can potentially be exposed by separately analyzing the risks of mortality associated with individuals who died before a given skeletal lesion healed (i.e., “active” lesions) and individuals who died sometime after the healing process was completed (*51*, *52*). Research by DeWitte (*53*), for instance, highlights the importance of considering periosteal lesion activity when evaluating heterogeneous frailty. In that study, Kaplan-Meier survival analysis indicated that individuals with healed periosteal lesions exhibited higher survivorship compared to individuals with active periosteal lesions and individuals with no visible periosteal lesions. In such studies, the stages of lesion activity or formation may provide additional information on differential frailty and resilience, beyond the conclusions that can be drawn from studies of lesion presence or absence and their associations with mortality or survivorship.

### Frailty as skeletal phenotype

Paleoepidemiological and bioarchaeological definitions—and consequent operationalizations—of frailty generally concentrate on frailty as increased risk of mortality relative to one’s age cohort (*7*). This definition and conceptualization arguably revitalized research that directly engaged with and considered the osteological paradox (*8*–*10*, *15*, *51*) and encouraged researchers to embrace issues of “healthy” lesions, selective mortality, and hidden heterogeneity. By contrast, other researchers have drawn from modern clinical and geriatric medicine for possible frailty definitions and applications. Frailty in the living (section Frailty in the living), for example, describes the impacts of cumulative stress to the body and considers multiple body systems in its analysis (*1*, *23*, *54*). These frailty biomarkers and subsequent cumulative indices are used to identify mortality and morbidity risks, oftentimes for risk mitigation in built environments (*2*). While frailty correlates directly with increased mortality and morbidity, it inherently contributes to discussions of resilience as frailty correlates with natural aging processes. Therefore, by conceptualizing frailty in the past in such a way, as a phenotype of both cumulative stress and resilience, we have the potential to tease apart this complicated topic in bioarchaeology.

Although survivorship and hazard analyses have brought the discipline to a more sophisticated level of statistical analysis and interpretation, these assessments, while robust, often analyze lesions or conditions singularly [for exception, see (*16*)]. By contrast, indexical approaches, on both population and individual scales, attempt to provide cumulative measures of frailty by incorporating multiple skeletal and dentoalveolar indicators (e.g., the absence/presence/severity of lesions and growth proxies). Steckel and Rose, through the Global History of Health Project (GHHP) (*55*), were notably the first to develop stress indices (numeric measures) for past populations based on seven attributes. Observable attributes are individually scored on a 0 to 100 scale, “worst” to “best” health, converted according to age-specific rates in designated age categories, and adjusted by the relative proportion of person-years lived within specified age groups. The overall index is an equally weighted average of these attributes. Although the GHHP codebook (*56*) was adopted and is still used as a guideline for data collection in many bioarchaeological studies, the health index has not been as readily applied to frailty research and critiqued for its application to incomplete skeletal samples (*57*).

More recent operationalizations of frailty in bioarchaeology have stemmed directly from human biology and clinical medicine, instituting both definitions and frameworks of frailty as a phenotype. Crews and colleagues (*12*–*14*) developed a skeletal frailty index (SFI) similar to allostatic load and modern frailty indices, whereby unweighted biomarkers indicative of stress episodes, acute or chronic, are scored as high (“1”) or low (“0”) frailty based on reported mortality/morbidity correlations and totaled to create a cumulative frailty score. While the original SFI use 13 biomarkers, metric and nonmetric, from the standard paleopathological suite, subsequent studies showed how SFIs with fewer biomarkers could yield similarly informative and representative results (*12*, *58*). Thus far, SFIs have been used to evaluate differences in cumulative frailty between medieval London monastic and nonmonastic communities (*13*), between ascribed socioeconomic status groups in postmedieval industrializing London (*17*), and between estimated sexes in medieval Poland (*58*).

Zedda and colleagues (*59*) subsequently proposed a biological index of frailty (BIF) that incorporated the same biomarkers as in the original (13-biomarker) SFI. Unlike the original and subsequent SFI iterations, the BIF weights individual biomarkers based on the odds ratio of dying prematurely through logit modeling, wherein age is the dependent variable and biomarker score is the independent variable. While the weighting of biomarkers was recognized as a limitation of the original SFI (*13*), the BIF is otherwise not a drastic alteration in frailty methods. One of the potential benefits of the BIF is that it increases the sample size for frailty evaluation by reducing the assessable biomarkers per individual. In their follow-up to the original SFI publication, Marklein and Crews (*12*) demonstrated the efficacy of reducing the number of biomarkers to increase sample representativeness and showed how a 4-biomarker SFI offered comparable results to 11-biomarker frailty indices. While the BIF does increase sample inclusivity and size, representativeness of frailty is not comparable between individuals and samples. For example, one of the purported benefits of the BIF is the inclusion of incomplete or fragmentary individuals. However, if an incomplete individual can only be scored for periosteal lesions, trauma, and osteoarthritis (OA), then this individual’s BIF score is essentially not comparable to the BIF score of an individual scored for linear enamel hypoplasia (LEH), periodontal disease (PD), periosteal lesions, trauma, and OA. This index, while attempting to be inclusive of more individuals and fragmentary/commingled samples, is repeating some of the limitations of the GHHP health index (*57*). Nonetheless, in many skeletal collections, where preservation and representation are poor, this index theoretically estimates a minimum value of frailty, despite missing data.

The methodological exercise we present in this paper, which combines risk of mortality with cumulative indices, showcases the strength of incorporating both approaches into a singular robust measure of frailty. First, we evaluate the cumulative frailty concept (SFI) and its relation to risk of mortality for adult individuals (Results). Subsequently, we develop and build population-specific indices—frailty (i.e., increased risk of mortality and decreased survivorship) and resilience (i.e., decrease risk of mortality and increased survivorship)—from individual skeletal biomarkers (Materials and Methods). From these new frailty and resilience scores, tabulated from these new indices, we explore in Discussion the female-male morbidity-mortality paradox in a medieval London context.

## RESULTS

### Analysis of SFIs

Before developing new frailty indices based on survival and hazards analyses, we examined the relationships between SFIs constructed following Marklein and Crews’ original approach (referred to here as the 5-biomarker and 6-biomarker SFIs) and survivorship and risk of mortality. The results of Kaplan-Meier analysis of the 5-biomarker SFI [LEH, PD, OA, periosteal new bone formation (PNBF), and trauma] and the 6-biomarker SFI (LEH, PD, OA, PNBF, trauma, and femur length) using presence/absence (binary) data are shown in table S1. Kaplan-Meier analyses of the SFI values using severity/lesion activity (scalar) data are uninformative given the sheer number of values produced using the scalar approach (in contrast to just six values using binary data), and thus results are not shown here. For each binary SFI, there is a significant association between SFI value and survivorship. For the 5-biomarker SFI, the highest estimated survivorship is found for those with no biomarkers (SFI = 0) followed by those with all five biomarkers present (SFI = 5). In general, survivorship drops from SFI = 0 to SFI = 2 and increases from SFI = 2 to SFI = 5. For the 6-biomarker SFI, estimated survivorship is highest for those people with five biomarkers present (SFI = 5; the maximum score observed in this sample), followed by those with four biomarkers present; however, survivorship does not increase consistently with SFI value. For both binary SFIs, the fact that relatively high survivorship is observed at both ends of the range of SFI values suggests that there is variation in how well each individual biomarker reflects frailty or resilience. As detailed in Materials and Methods below, this finding, combined with information from previous studies regarding the age patterns of some of the biomarkers included here (e.g., OA tends to increase in frequency with age) motivated us to examine the associations between each individual biomarker and survivorship to identify which biomarkers could most appropriately be grouped together in novel indices.

The results of Cox proportional hazards analysis of the 5-biomarker and 6-biomarker SFIs are shown in table S2. For the binary 5-biomarker SFI, there is no significant association between SFI value and hazards of death when we do not specify a reference. However, when we specify SFI = 0 as the reference, all SFI values 1 to 5 are associated with higher hazards of death compared to SFI = 0, although not all of these results are significant (please refer to Materials and Methods regarding our approach to and justification for specifying a reference in the Cox analyses). Using scalar data (without specifying a reference given that there are too many scalar SFI values to do so informatively), Cox analysis of the 5-biomarker SFI reveals significantly lower hazards with increased SFI values. For the binary 6-biomarker SFI (using binary data and without specifying a reference), the estimated hazard decreases significantly with increasing SFI value. When we specify SFI = 0 as the reference, most of the associations between individual SFI values and hazards of death are not significant. Using scalar data (without specifying a reference), Cox analysis of the 6-biomarker SFI reveals significantly lower hazards with increased SFI values. As with the Kaplan-Meier results, these findings combined suggest variation in what each biomarker (or severity/activity thereof) reflects regarding frailty. As was the case with the survival analyses of the indices, this variation further motivated us to evaluate how each individual biomarker was associated with hazards of death prior to constructing new indices.

The results of the Gompertz analysis using binary data are shown in table S3. Using binary data, the Gompertz results suggest that the parameter representing the effect of the SFI covariate did not significantly affect risk of death. However, the Gompertz analysis of the scalar SFI values is somewhat uninformative, given that the results do not indicate if the observed relationship is consistent across all levels or scales. Consequently, we direct the reader to the more informative results produced by the Cox proportional hazards analysis using the scalar data, described above.

### Analysis of individual biomarkers

The results of Kaplan-Meier analysis of the presence/absence of the individual biomarkers are shown in Table 1. The presence of LEH and short femur length is each associated with significantly lower survivorship; the presence of PNBF and OA is each associated with significantly higher survivorship; there are no significant associations between survivorship and the presence of cribra orbitalia, osteoporosis, PD, rickets, short tibiae, or trauma. Table 1 also includes the results of Kaplan-Meier analysis of the severity or activity (as relevant) of LEH, PNBF, PD, cribra orbitalia, and trauma. Survivorship significantly and consistently decreases with increasing numbers of LEH. For cribra orbitalia, individuals can be organized from lowest to highest survivorship as follows: stage 3, stage 4, stage 2, absence, and stage 1 (*60*). There is significant variation in survivorship based on the activity of PNBF; specifically, as has been observed previously (*53*), individuals can be organized from lowest to highest survivorship as follows: active, absence, mixed, and healed PNBF. With respect to PD, individuals can be organized from lowest to highest survivorship as follows: 1, absence, 2, and 3; however, there is substantial crossing-over of the survival functions, which can make patterns difficult to interpret. We also observe no significant association between trauma with secondary infection and survivorship.

**Table 1.** Kaplan-Meier survival analysis of the presence versus absence and severity/activity of individual biomarkers. Mean survival times (mean ages at death) in years are shown with 95% confidence intervals in parentheses. “Short” femoral and tibial lengths defined as 2 SDs below sex-specific average. LEH, linear enamel hypoplasia; OA, osteoarthritis; PD, periodontal disease; PNBF, periosteal new bone formation; CI, confidence interval; *P*, *P* value; *n*, number of cases; N.A., not applicable.

Biomarker	Presence versus absence	Mean age at death (95% CI)	*P*	Activity or severity	Mean age at death (95% CI)	*P*
LEH (*n* = 1439)	Absent	41.23 (39.70–42.76)	<0.001***	Absent	41.23 (39.70–42.76)	<0.001***
	Present	32.53 (31.47–33.59)		1 defect	37.53 (33.56–41.50)	
				2 defects	35.11 (33.28–36.93)	
				3 defects	29.38 (26.58–32.17)	
				4 defects	29.90 (28.44–31.36)	
Cribra orbitalia (*n* = 858)	Absent	35.04 (33.69–36.40)	0.79	Absent	35.04 (33.69–36.40)	0.049**
	Present	34.50 (32.57–36.43)		Stage 1	37.75 (34.84–40.66)	
				Stage 2	31.72 (28.60–34.85)	
				Stage 3	30.15 (25.38–34.92)	
				Stage 4	30.79 (24.39–37.19)	
OA (*n* = 1443)	Absent	35.24 (34.31–36.17)	<0.001***	N.A.		
	Present	48.02 (44.48–51.56)				
Osteoporosis (*n* = 1257)	Absent	36.01 (35.02–37.00)	0.27	N.A.		
	Present	47.66 (18.15–77.17)				
PD (*n* = 948)	Absent	33.44 (30.55–36.33)	0.16	Absent	33.44 (30.55–36.33)	<0.001***
	Present	35.84 (34.69–36.98)		1	29.16 (26.68–31.64)	
				2	34.36 (32.47–36.24)	
				3	39.22 (37.56–40.88)	
PNBF (*n* = 973)	Absent	34.04 (32.47–35.60)	0.001**	Absent	34.04 (32.47–35.60)	<0.001***
	Present	37.50 (36.07–38.94)		Healed	38.61 (36.96–40.27)	
				Mixed	35.19 (32.23–38.14)	
				Active	22.42 (17.17–27.68)	
Rickets (*n* = 1020)	Absent	36.39 (35.32–37.47)	0.20	N.A.		
	Present	27.38 (19.00–35.77)				
Short femur length (*n* = 674)	Average/tall	34.85 (33.68–36.02)	0.05*	N.A.		
	Short	27.94 (23.90–31.98)				
Short tibia length (*n* = 621)	Average/tall	34.74 (33.52–35.97)	0.49	N.A.		
	Short	32.60 (20.36–44.83)				
Trauma (*n* = 1443)	Absent	35.67 (34.65–36.69)	0.12	Absent	35.67 (34.65–36.69)	0.19
	Present	38.49 (36.47–40.52)		Trauma with no infection	38.89 (36.80–40.97)	
				Trauma with infection	32.68 (24.55–40.81)	

*P < 0.1.

**P < 0.05.

***P < 0.001.

The Cox proportional hazards analysis results of the binary biomarker data are shown in Table 2. These results indicate that the presence of LEH and short femur length is each associated with significantly higher hazards of death. Conversely, PNBF and OA are each associated with significantly lower hazards of death. There were no significant associations between hazard of death and the presence of cribra orbitalia, osteoporosis, rickets, PD, short tibia length, or trauma. Also shown in Table 2 are the results of Cox analyses using scalar data (available for LEH, PNBF, PD, cribra orbitalia, and trauma) for which we specify “absence” as the reference. These indicate significantly increased hazard of death with increasing numbers of LEH, significantly lower hazards for those with healed PNBF and higher hazards for those with active PNBF, significantly higher hazards for people with PD low severity (score = 1) but lower hazards for those with PD severity scores of 3, significantly higher hazards for people with cribra orbitalia scores equal to 2, and a significantly lower hazard for people with trauma but no detectable infection.

**Table 2.** Cox proportional hazards analysis of the presence versus absence and severity/activity of individual biomarkers. Individuals without the relevant biomarker are the reference group for each comparison. “Short” femoral and tibial lengths defined as 2 SDs below sex-specific average. LEH, linear enamel hypoplasia; OA, osteoarthritis; PD, periodontal disease; PNBF, periosteal new bone formation; *P*, *P* value; CI, confidence interval; *n*, number of cases; N.A., not applicable.

Biomarker	Presence versus absence	Exp(β) (95% CI)	*P*	Activity or severity	Exp(β) (95% CI)	*P*
LEH (*n* = 1439)	Present	1.64 (1.47–1.82)	<0.001***	1 defect	1.27 (0.97 0 1.64)	0.07*
				2 defects	1.41 (1.23–1.61)	<0.001***
				3 defects	2.15 (1.71–2.71)	<0.001***
				4 defects	1.95 (1.71–2.23)	<0.001***
Cribra orbitalia (*n* = 858)	Present	1.02 (0.89–1.18)	0.79	Stage 1	0.87 (0.725–1.04)	0.13
				Stage 2	1.25 (0.99–1.57)	0.06*
				Stage 3	1.27 (0.93–1.76)	0.13
				Stage 4	1.13 (0.70–1.82)	0.62
OA (*n* = 1443)	Present	0.56 (0.46–0.69)	<0.001***	N.A.		
Osteoporosis (*n* = 1257)	Present	0.53 (0.17–1.65)	0.27	N.A.		
PD (*n* = 948)	Present	0.89 (0.75–1.05)	0.16	1	1.40 (1.11–1.77)	0.004**
				2	0.96 (0.79–1.17)	0.69
				3	0.75 (0.63–0.90)	0.002**
PNBF (*n* = 973)	Present	0.81 (0.71–0.92)	0.001**	Healed	0.77 (0.67–0.88)	<0.001***
				Mixed	0.90 (0.73–1.1)	0.29
				Active	2.64 (1.45–4.81)	0.002**
Rickets (*n* = 1020)	Present	2.1 (0.66–6.39)	0.21	N.A.		
Short femur length (*n* = 674)	Short	1.89 (0.98–3.66)	0.06*	N.A.		
Short tibia length (*n* = 621)	Short	1.28 (0.64–2.57)	0.49	N.A.		
Trauma (*n* = 1443)	Present	0.90 (0.78–1.03)	0.12	Trauma with no infection	0.89 (0.77–1.02)	0.09*
				Trauma with infection	1.13 (0.69–1.84)	0.64

*P < 0.1.

**P < 0.05.

***P < 0.001.

The Gompertz analysis results of the binary biomarker data are shown in Table 3. These results indicate that the presence of LEH is associated with significantly higher risks of death. In contrast, PNBF and OA are each significantly associated with lower risks of death. There were no significant associations between risk of death and PD, cribra orbitalia, trauma, osteoporosis, rickets, short femur length, or short tibia length. As with the analysis of the original SFI values presented above, the results of Gompertz analyses using scalar data are not considered as informative as the Cox analysis results when applied to scalar data (i.e., the results do not indicate if the observed relationship is consistent across all levels or scales) and were not considered in the construction of the revised scalar indices described below.

**Table 3.** Gompertz analysis of the presence versus absence of individual biomarkers. Results include maximum likelihood estimates of the effect of the biomarker presence covariate (with 95% confidence intervals in parentheses) and LRTs of *H*_0_: Effect of biomarker presence covariate = 0 for the individual biomarkers. “Short” femoral and tibial lengths defined as 2 SDs below sex-specific average. LEH, linear enamel hypoplasia; OA, osteoarthritis; PD, periodontal disease; PNBF, periosteal new bone formation; CI, confidence interval; *P*, *P* value; *n*, number of cases.

Biomarker	Covariate estimate (95% CI)	LRT	*P*
LEH (*n* = 1439)	0.470 (0.358–0.577)	76.392	<0.001***
Cribra orbitalia (*n* = 858)	0.021 (−0.171–0.203)	0.09	0.764
OA (*n* = 1443)	−0.537 [−0.864–(−0.241)]	32.204	<0.001***
Osteoporosis (*n* = 1257)	−0.514 (−3.114–0.893)	0.942	0.332
PD (*n* = 948)	−0.060 (−0.175–0.052)	0.486	0.486
PNBF (*n* = 973)	−0.171 [−0.311–(−0.038)]	6.978	0.008**
Rickets (*n* = 1020)	0.711 (−1.890–2.117)	1.208	0.272
Short femur length (*n* = 674)	0.607 (−0.673–1.506)	2.702	0.100
Short tibia length (*n* = 621)	0.076 (−1.299–1.021)	0.044	0.834
Trauma (*n* = 1443)	−0.102 (−0.308–0.091)	2.218	0.136

*P < 0.1.

**P < 0.05.

***P < 0.001.

**Fig. 1.**
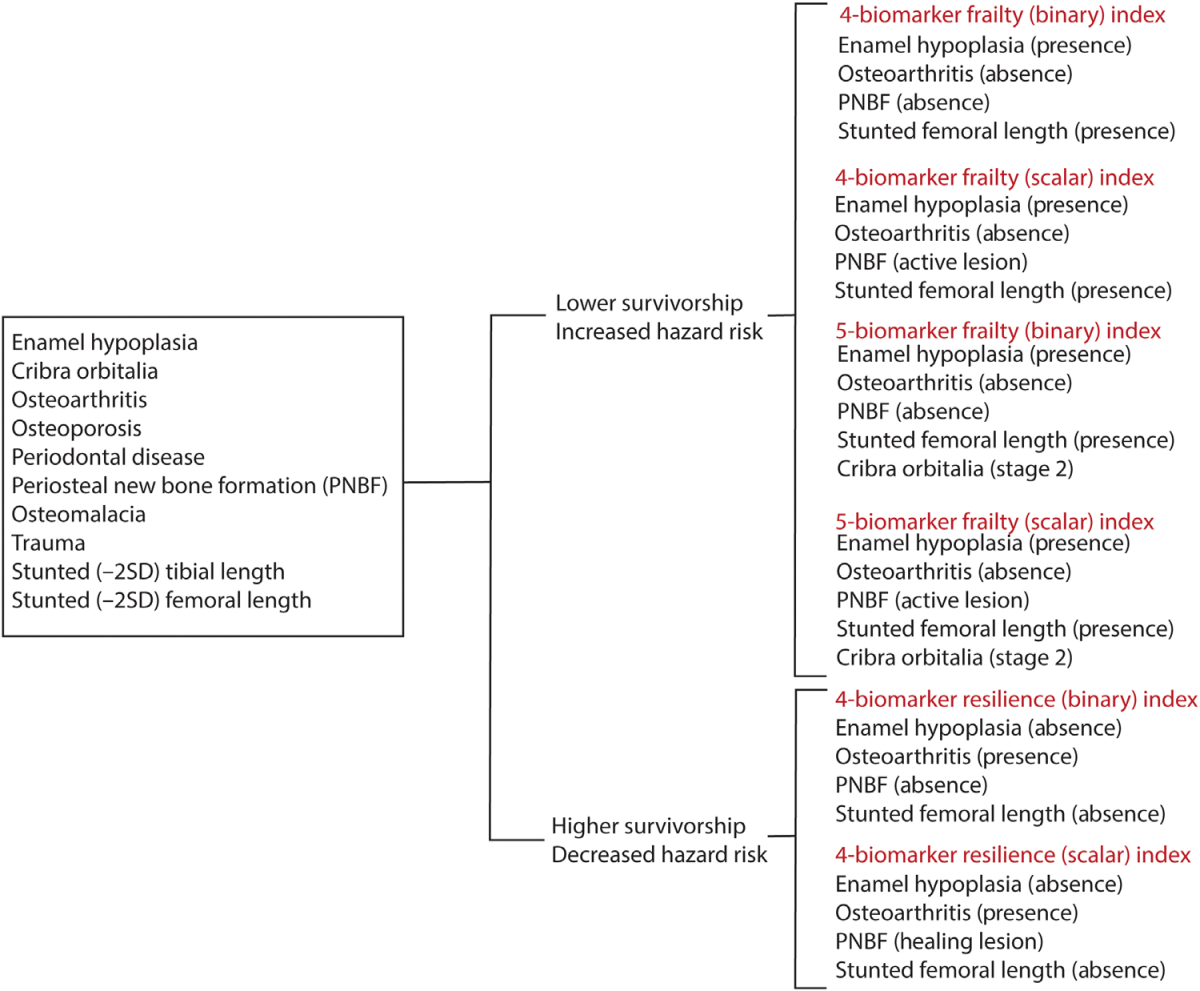
Revised frailty and resilience indices. Six new frailty and resilience indices were constructed using data on the presence versus absence (binary) or severity/activity (scalar) of biomarkers associated with frailty (lower survivorship and increased hazard risk) or resilience (higher survivorship and decreased hazard risk). PNBF, periosteal new bone formation; SD, standard deviation.

On the basis of these results, we created revised indices by selectively combining biomarkers that had significant associations with survivorship and risks of death and for which the survival and hazards analysis were consistent (i.e., high hazards of death and low survivorship, or vice versa) (Fig. 1). Thus, we exclude trauma from the revised indices because the results of survival and hazards analyses for this biomarker are not in agreement. Furthermore, we exclude PD from the revised indices because, as mentioned above, the substantial crossing-over of survival functions can interfere with interpretations of findings. Our ultimate goal is to more accurately capture signals of frailty (as measured by higher risks of mortality and lower survivorship) or resilience (as measured by lower risks of mortality and higher survivorship). We thus refer to these revised indices as the (i) frailty indices and (ii) resilience indices. There are two iterations of each of these indices: one that includes four biomarkers (LEH, PNBF, OA, and femur length) and one that also includes cribra orbitalia for a total of five biomarkers.

Four-biomarker frailty indices—binary and scalar—were constructed from LEH, PNBF, OA, and short femoral length data. Unlike the original SFI, where the presence of a pathological condition garnered a high (“1”) frailty score, frailty scores for the new frailty index were determined by higher survivorship and lower hazard of mortality. For the binary frailty index, the presence of LEH and femoral stunting all received high (“1”) frailty scores, whereas the absence of OA and PNBF received a high frailty score. Consequently, an individual with LEH, short femora, and no OA and PNBF would have the highest frailty index score, “4,” whereas an individual with no LEH, average femoral length, and OA and PNBF presence would have the lowest frailty index score, “0.” The same scoring criteria were used for LEH, femoral length, and OA in the scalar frailty index; however, PNBF was scored for high frailty only when the individual had an active lesion at the time of death. Five-biomarker frailty indices—binary and scalar—included cribra orbitalia as cribra orbitalia was associated with higher hazards of death. In these frailty indices, cribra orbitalia was scored for high frailty (“1”) when an individual exhibited large and small isolated foramina [stage 2, (*60*)]. Therefore, individuals with no LEH, PNBF presence, average femoral length, OA presence, and any cribra orbitalia stage not 2 (i.e., no cribra orbitalia, stage 1, stage 3, and stage 4) would have the lowest frailty index score, “0,” and individuals with stage 2 cribra orbitalia, LEH presence, no PNBF, short femoral length, and OA absence would have the highest frailty score, “5.” For scalar frailty indices, identical criteria were used for LEH, femoral, OA, and cribra orbitalia, but only active PNBF was scored as high frailty.

As with frailty indices, resilience indices include LEH, PNBF, OA, and femoral lengths. However, resilience indices were constructed with biomarkers that demonstrated significant evidence of higher survivorship and lower risk of mortality. For example, the absence of LEH, presence of PNBF, normal (i.e., not stunted) femoral length, and presence of OA were all associated with higher survivorship and lower hazards of death and thus received a high resilience score (“1”); by contrast, the presence of LEH, absence of PNBF, stunted femoral length, and absence of OA were associated with higher hazards of death and thus received low resilience scores (“0”). When lesion severity/activity was considered, healed PNBF was associated with high resilience (“1”). Subsequently, for the binary resilience index, an individual without LEH, with PNBF, normal femoral length, and OA would have the highest resilience index score, “4,” and the opposite lesion presence would yield a low resilience index score, “0.” For the scalar resilience index, healed PNBF was scored as high resilience.

### Analysis of revised frailty and resilience indices

In the analyses of the original SFI and the individual biomarkers, we used nonparametric (Kaplan-Meier), semiparametric (Cox proportional hazards), and fully parametric (Gompertz hazards) approaches to confirm if any observed trend was accurate and not a statistical artifact. Given the consistency and informativeness of the results produced in the analyses of the original SFI and the individual biomarkers, we simplified our analytical approach to assess the performance of the revised frailty and resilience indices using only nonparametric (Kaplan-Meier) and semiparametric (Cox proportional hazards) methods.

The results of Kaplan-Meier analysis of the revised 4-biomarker and 5-biomarker indices are shown in Table 4. For the revised frailty indices, the results indicate that higher index values are consistently associated with significantly lower survivorship, and survivorship decreases consistently with increasing index values. For the resilience indices, higher values are significantly associated with higher survivorship, and survivorship increases consistently with increasing index values.

**Table 4.** Kaplan-Meier analysis of revised 4-biomarker and 5-biomarker frailty and resilience indices. Mean survival times (mean ages at death) in years are shown with 95% confidence intervals in parentheses. *P*, *P* value; CI, confidence interval; *n*, number of cases; N.A., not applicable.

Index	Index score	Mean age at death (95% CI)	*P*
4-biomarker frailty (binary) (*n* = 581)	0	43.07 (35.97–50.16)	<0.001***
	1	41.12 (38.41–43.84)	
	2	33.93 (32.19–35.67)	
	3	29.15 (27.28–31.01)	
	4	23.79 (15.95–31.62)	
4-biomarker frailty (scalar) (*n* = 581)	0	49.88 (43.01–56.74)	<0.001***
	1	38.23 (36.18–40.29)	
	2	31.20 (29.81–32.60)	
	3	22.63 (18.78–26.48)	
	4	N.A.	
5-biomarker frailty (binary) (*n* = 343)	0	44.93 (34.75–55.12)	<0.001***
	1	41.55 (36.79–46.32)	
	2	33.60 (31.55–35.65)	
	3	29.25 (27.29–31.22)	
	4	26.59 (23.05–30.13)	
	5	N.A.	
5-biomarker frailty (scalar) (*n* = 343)	0	51.81 (39.98–63.65)	<0.001***
	1	39.63 (36.00–43.25)	
	2	31.35 (29.81–32.88)	
	3	26.26 (24.12–28.40)	
	4	N.A.	
	5	N.A.	
4-biomarker resilience (binary) (*n* = 581)	0	23.79 (15.95–31.62)	<0.001***
	1	29.15 (27.28–31.01)	
	2	33.93 (32.19–35.67)	
	3	41.12 (38.41–43/84)	
	4	43.07 (35.97–50.16)	
4-biomarker resilience (scalar) (*n* = 581)	0	23.79 (15.95–31.62)	<0.001***
	1	29.36 (27.63–31.10)	
	2	35.55 (33.71–37.39)	
	3	41.02 (37.99–44.04)	
	4	43.30 (35.42–51.19)	

*P < 0.1.

**P < 0.05.

***P < 0.001.

The Cox hazards analysis results are shown in Table 5. The analyses reveal consistent increases in hazards of death with increasing frailty index values for the 4-biomarker and 5-biomarker (binary and scalar) frailty indices; these results are generally significant, with a few exceptions as seen in Table 5. For the resilience indices (binary and scalar), the results indicate consistent decreases in hazards of death with increasing index values; as with the frailty indices, the results are generally significant, with a few exceptions as seen in Table 5.

**Table 5.** Cox proportional hazards analysis of revised 4-biomarker and 5-biomarker frailty and resilience indices. Individuals with index scores of 0 are the reference group for each comparison. *P*, *P* value; CI, confidence interval; *n*, number of cases; N.A., not applicable.

Index	Index score	Exp(β) (95% CI)	*P*
4-biomarker frailty (binary) (*n* = 581)	1	1.11 (0.69–1.78)	0.68
	2	1.71 (1.07–2.73)	0.03**
	3	2.55 (1.58–4.13)	<0.001***
	4	5.52 (1.63–18.76)	0.006**
4-biomarker frailty (scalar) (*n* = 581)	1	1.65 (1.13–2.42)	0.01**
	2	2.74 (1.87–4.02)	<0.001***
	3	7.77 (3.76–16.07)	<0.001***
	4	N.A.	N.A.
5-biomarker frailty (binary) (*n* = 343)	1	1.16 (0.52–2.56)	0.72
	2	1.85 (0.86–3.95)	0.11
	3	2.77 (1.29–5.96)	0.009**
	4	3.85 (1.56–9.50)	0.004**
	5	N.A.	N.A.
5-biomarker frailty (scalar) (*n* = 343)	1	1.58 (0.79–3.18)	0.20
	2	2.85 (1.45–5.58)	0.002**
	3	5.28 (2.51–11.10)	<0.001***
	4	N.A.	N.A.
	5	N.A.	N.A.
4-biomarker resilience (binary) (*n* = 581)	1	0.46 (0.15–1.45)	0.19
	2	0.31 (0.10–0.97)	0.04**
	3	0.20 (0.06–0.63)	0.006**
	4	0.18 (0.05–0.62)	0.006**
4-biomarker resilience (scalar) (*n* = 581)	1	0.45 (0.14–1.40)	0.17
	2	0.28 (0.09–0.88)	0.029**
	3	0.20 (0.06–0.64)	0.007**
	4	0.18 (0.05–0.62)	0.007**

*P < 0.1.

**P < 0.05.

***P < 0.001.

### Spearman’s rho correlation results

A Spearman’s rank-order correlation was used to assess the degree to which each biomarker contributes to the revised indices (i.e., the strength of the association between each biomarker and a given individual’s frailty or resilience index value) within the total sample and the female and male subsamples. All of the Spearman’s correlations were computed in SPSS version 29. For 4-biomarker frailty, 4-biomarker resilience, and 5-biomarker frailty indices, females and males exhibit similar correlations between individual biomarkers and index values. For nearly all analyses, correlations between biomarkers and frailty and resilience indices are significant, which is to be expected as indices are constructed from these biomarkers (Table 6). We outline results for the overall sample below as male and female subsamples yielded comparable correlations.

**Table 6.** Spearman’s correlations. Results compare 5-biomarker frailty index (binary and scalar) values, 4-biomarker frailty index (binary and scalar) values, and 4-biomarker resilience index (binary and scalar) values with high (“1”) frailty or resilience biomarkers, respectively. LEH, linear enamel hypoplasia; OA, osteoarthritis; PD, periodontal disease; PNBF, periosteal new bone formation; −2SD, 2 SDs below sex-specific average; rho, correlation coefficient; *P*, *P* value; *n*, number of cases.

		All adults		Estimated male		Estimated female	
		5-biomarker frailty (binary)	5-biomarker frailty (scalar)	5-biomarker frailty (binary)	5-biomarker frailty (scalar)	5-biomarker frailty (binary)	5-biomarker frailty (scalar)
LEH (absent)	rho	0.551***	0.740***	0.550***	0.754***	0.556***	0.731***
	*P*	<0.001	<0.001	<0.001	<0.001	<0.001	<0.001
	*n*	343	343	172	172	171	171
PNBF (present)	rho	0.702***		0.790***		0.614***	
	*P*	<0.001		<0.001		<0.001	
	*n*	343		172		171	
PNBF (active)	rho		0.223***		0.212***		0.231***
	*P*		<0.001		0.005		0.002
	*n*		343		172		171
Cribra orbitalia (stage 2)	rho	0.377***	0.495***	0.347***	0.462***	0.405***	0.523***
	*P*	<0.001	<0.001	<0.001	<0.001	<0.001	<0.001
	*n*	343	343	172	172	171	171
OA (present)	rho	0.359***	0.451***	0.359***	0.446***	0.364***	0.455***
	*P*	<0.001	<0.001	<0.001	<0.001	<0.001	<0.001
	*n*	343	343	172	172	171	171
Femoral length (−2SD)	rho	0.173**	0.175**	0.173**	0.111	0.172**	0.231**
	*P*	0.001	0.001	0.023	0.146	0.024	0.002
	*n*	343	343	172	172	171	171
		All adults		Estimated male		Estimated female	
		**4-biomarker frailty (binary)**	**4-biomarker frailty (scalar)**	**4-biomarker frailty (binary)**	**4-biomarker frailty (scalar)**	**4-biomarker frailty (binary)**	**4-biomarker frailty (scalar)**
LEH (absent)	rho	0.700***	0.907***	0.694***	0.919***	0.707***	0.896***
	*P*	<0.001	<0.001	<0.001	<0.001	<0.001	<0.001
	*n*	581	581	296	296	285	285
PNBF (absent)	rho	0.673***		0.692***		0.655***	
	*P*	<0.001		<0.001		<0.001	
	*n*	581		296		285	
PNBF (active)	rho		0.181***		0.160***		0.200***
	*P*		<0.001		0.006		0.001
	*n*		581		296		285
OA (present)	rho	0.363***	0.436***	0.399***	0.445***	0.326***	0.426***
	*P*	<0.001	<0.001	<0.001	<0.001	<0.001	<0.001
	*n*	581	581	296	296	285	285
Femoral length (−2SD)	rho	0.184***	0.181***	0.178**	0.135**	0.192**	0.226***
	*P*	<0.001	<0.001	0.002	0.02	0.001	<0.001
	*n*	581	581	296	296	285	285
		All adults		Estimated male		Estimated female	
		**4-biomarker resilience (binary)**	**4-biomarker resilience (scalar)**	**4-biomarker resilience (binary)**	**4-biomarker resilience (scalar)**	**4-biomarker resilience (binary)**	**4-biomarker resilience (scalar)**
LEH (present)	rho	0.700***	0.681***	0.694***	0.681***	0.707***	0.682***
	*P*	<0.001	<0.001	<0.001	<0.001	<0.001	<0.001
	*n*	581	581	296	296	285	285
PNBF (present)	rho	0.673***		0.692***		0.655***	
	*P*	<0.001		<0.001		<0.001	
	*n*	581		296		285	
PNBF (healed)	rho		0.666***		0.700***		0.628***
	*P*		<0.001		<0.001		<0.001
	*n*		581		296		285
OA (present)	rho	0.363***	0.387***	0.399***	0.411***	0.326***	0.363***
	*P*	<0.001	<0.001	<0.001	<0.001	<0.001	<0.001
	*n*	581	581	296	296	285	285
Average femoral length	rho	0.184***	0.177***	0.178**	0.166**	0.192**	0.187**
	*P*	<0.001	<0.001	0.002	0.004	0.001	0.002
	*n*	581	581	296	296	285	285

*P < 0.1.

**P < 0.05.

***P < 0.001.

The results indicate that LEH presence, among all biomarkers, exhibits significantly high correlations with frailty or resilience index values in both 4-biomarker binary and scalar frailty indices (rho = 0.700, rho = 0.907) and resilience indices (rho = 0.700, rho = 0.681) and 5-biomarker frailty binary and scalar indices (rho = 0.551, rho = 0.740). PNBF presence is also significantly correlated with 4-biomarker and 5-biomarker frailty indices, rho = 0.673 and rho = 0.702, respectively, while the absence of PNBF correlates significantly with 4-biomarker resilience indices (rho = 0.673). Although cribra orbitalia (stage 2) correlates with 5-biomarker binary and scalar frailty indices, the correlations are markedly lower, rho = 0.377 (binary) and rho = 0.495 (scalar), than LEH and PNBF. These results are similar to the absence of OA, which contributes moderately to 5-biomarker binary and scalar frailty indices, rho = 0.359 and rho = 0.451, and 4-biomarker binary and scalar frailty indices, rho = 0.363 and rho = 0.436, respectively. The presence of OA also contributes moderately to 4-biomarker binary (rho = 0.363) and scalar (rho = 0.387) resilience indices. Last, of all the biomarkers, shorter femoral length and “normal” femoral length correlates weakly with 4-biomarker and 5-biomarker frailty indices and 4-biomarker resilience indices, rho values ranging 0.173 to 0.184, and shorter femoral lengths for males do not correlate significantly with the 5-biomarker scalar frailty index.

### Comparison of frailty and resilience indices in medieval London

To address a larger question of frailty differentials by sex, we compared distributions of frailty and resilience scores, based on the revised frailty and resilience indices, between estimated adult females and males. As previously described, individuals with the observable biomarkers were scored for four frailty indices (4-biomarker and 5-biomarker and binary and scalar versions of each) and two resilience indices (4-biomarker and binary and scalar). Binary logistic regressions were used to compare frailty and resilience scores between sexes and consider age as a possible confounding variable. Analyses were conducted in SPSS version 29. Table 7 shows the results of frailty and resilience scores by sex. Frailty scores for both 4-biomarker and 5-biomarker indices were nearly identical between males and females; similarly, resilience scores based on 4-biomarker and 5-biomarker were not significantly different between females and males. According to logistic regression, no significant differences were observed between sexes, and age was not a confounding variable (Table 8). For the 5-biomarker frailty index subsample (*n* = 343), females yielded an average age (SD) of 32.7 (±13.6) years and males 33.5 (±13.2) years; for 4-biomarker frailty and resilience samples (*n* = 581), average age and SD for females and males were 34.4 (±15.8) years and 35.6 (±14.9) years, respectively.

**Table 7.** Distributions of frailty and resilience index values by estimated sex. SD, standard deviation; *n*, number of cases; *n*_f_, number of female cases; *n*_m_, number of male cases.

Index	Estimated female Mean (SD)	*n*_f_	Estimated male Mean (SD)	*n*_m_
5-biomarker frailty index (binary)	2.36 (0.064)	171	2.43 (0.052)	172
5-biomarker frailty index (scalar)	1.84 (0.052)	171	1.85 (0.045)	172
4-biomarker frailty index (binary)	2.01 (0.046)	285	2.07 (0.043)	296
4-biomarker frailty index (scalar)	1.49 (0.037)	285	1.49 (0.036)	296
4-biomarker resilience index (binary)	1.99 (0.046)	285	1.93 (0.043)	296
4-biomarker resilience index (scalar)	3.84 (0.067)	285	3.84 (0.066)	296

**Table 8.** Binary logistic regression comparing index scores and age (independent variables) with estimated sex (dependent variable). Note the lack of significance in all outputs. No strong correlations (rho > 0.50) were observed between age and index scores. β, regression coefficient; SE, standard error; df, degrees of freedom; CI, confidence interval.

Variables in the equation	β	SE	Wald	df	*P*	Exp(β)	95% CI for Exp(β)	
							Lower	Upper
5-biomarker binary frailty index	0.022	0.139	0.025	1	0.875	1.022	0.779	1.342
Age	−0.004	0.009	0.276	1	0.599	0.996	0.979	1.012
Constant	0.094	0.497	0.035	1	0.851	1.098		
Variables in the equation	β	SE	Wald	df	*P*	Exp(β)	95% CI for Exp(β)	
							**Lower**	**Upper**
5-biomarker scalar frailty index	−0.080	0.184	0.192	1	0.661	0.923	0.644	1.322
Age	−0.006	0.009	0.528	1	0.468	0.994	0.977	1.011
Constant	0.352	0.530	0.441	1	0.507	1.421		
Variables in the equation	β	SE	Wald	df	*P*	Exp(β)	95% CI for Exp(β)	
							**Lower**	**Upper**
4-biomarker binary frailty index	0.044	0.108	0.166	1	0.684	1.045	0.846	1.290
Age	−0.004	0.006	0.574	1	0.449	0.996	0.985	1.007
Constant	0.030	0.336	0.008	1	0.930	1.030		
Variables in the equation	β	SE	Wald	df	*P*	Exp(β)	95% CI for Exp(β)	
							**Lower**	**Upper**
4-biomarker scalar frailty index	−0.042	0.141	0.086	1	0.769	0.959	0.727	1.265
Age	−0.006	0.006	0.934	1	0.334	0.994	0.983	1.006
Constant	0.219	0.345	0.401	1	0.527	1.244		
Variables in the equation	**β**	**SE**	**Wald**	**df**	***P***	**Exp(β)**	**95% CI for Exp(β)**	
							**Lower**	**Upper**
4-biomarker binary resilience index	−0.044	0.108	0.166	1	0.684	0.957	0.775	1.182
Age	−0.004	0.006	0.574	1	0.449	0.996	0.985	1.007
Constant	0.205	0.265	0.596	1	0.440	1.227		
Variables in the equation	β	SE	Wald	df	*P*	Exp(β)	95% CI for Exp(β)	
							**Lower**	**Upper**
4-biomarker scalar resilience index	−0.060	0.108	0.306	1	0.580	0.942	0.762	1.164
Age	−0.004	0.006	0.523	1	0.470	0.996	0.985	1.007
Constant	0.223	0.259	0.742	1	0.389	1.250		

## DISCUSSION

### Method efficacy

Methodologically, this paper endeavored to test the utility of the original SFI (which made no distinction between potential markers of frailty versus resilience) and the efficacy of combining two current frailty approaches in bioarchaeology, namely, frailty as risk of mortality and as a cumulative phenotype. Multiple SFIs were generated, while new frailty and resilience indices were constructed and subsequently tested.

Results from the original SFIs—a nonmetric 5-biomarker SFI and combined metric/nonmetric 6-biomarker SFI—demonstrated a clear correlation between higher SFI scores (i.e., “5”) and higher survivorship, but the relationship between SFI scores and hazards of death was less clear (tables S1 and S2). While individuals with no / of lesions exhibited the highest survivorship, which could reflect a confluence of both physiological health and cultural buffering, survivorship increased monotonically from low (“1”) to highest (“5”) frailty. This suggests that the original SFI results yielded concurrent frailty and resilience information as the lowest frailty scores (“0”) were associated with lowest mortality and highest survivorship (i.e., resilience) while the highest frailty scores (“5” and “6”) were associated with the second highest survivorship. As with previous frailty research on medieval London populations, these findings demonstrate how skeletal lesions oftentimes reflect resilience and occur at higher prevalence among older age groups (*12*, *14*). Consequently, it is proposed that the SFI, especially when it is composed of age-related conditions, be used as a measure of resilience or only be analyzed within age-specific groups to remove confounding age variables (*58*).

The novel frailty and resilience indices introduced in this study convey how survivorship and hazard data can be used to generate robust, cumulative measures of frailty and resilience within specific populations. Frailty indices were constructed using biomarkers that were associated with lower survivorship and higher hazards of death according to the absence, presence, or severity/activity of the condition among people from medieval London (specifically LEH, PNBF, OA, cribra orbitalia, and femoral length as a proxy for stature). While these indices included fewer biomarkers, those included were associated with higher risk of mortality in this population, rather than mortality risk data from the previous literature and other populations as was the case with the original SFI (*13*). Consequently, when we applied survivorship and hazards analyses to the frailty index scores, the results showed a significant correlation between high frailty scores and decreased survivorship/increased hazard, whereby individuals with lowest frailty scores lived longer than those with higher frailty scores (Tables 4 and 5). Similarly, resilience indices were constructed only using biomarkers that were associated with significantly higher survivorship and lower hazards (for the absence, presence, or severity/activity of a lesion). As with frailty indices, higher resilience index scores were associated with significantly higher survivorship/lower hazards (Tables 4 and 5). These indices are an improvement on the original SFI as biomarkers were selected specifically for the population under study and likely more reflective of what really constituted frailty in the medieval population. This approach of selecting biomarkers based on such data from the population under study aligns with emerging anthropological engagement with theoretical frameworks of “local biologies” or “situated biologies” (*61*, *62*). Ultimately, our results suggest that context-specific and population-specific frailty and resilience indices are a parsimonious solution for incorporating the informativeness and strengths of both hazard-based and cumulative phenotypic approaches to skeletal frailty.

### Early life stress

When we evaluated the revised frailty and resilience indices, we found that higher frailty index values were associated with higher hazards of death and lower survivorship, whereas higher resilience index values were associated with lower hazards of death and higher survivorship. This result suggests that the revised indices are useful measures of frailty and resilience in the context of medieval London. When Spearman’s correlations were used to assess the degree to which each biomarker correlates with the revised indices, LEH presence most strongly contributes to high frailty or resilience index values. Given that LEH form on the permanent mandibular canines in response to infection, malnutrition, and trauma prior to ~6 years of age (*63*), the consistently strong association between LEH presence and the frailty and resilience index values indicates that early life health insults had a pronounced effect on cumulative patterns of frailty and resilience across the life course in individuals living in medieval London.

The Developmental Origins of Health and Disease framework (DOHaD, also known as the Barker, fetal programming, or fetal origins hypotheses) recognizes the link between early life environments and health outcomes in later stages of the life course and has been studied in great detail in medical and biological sciences (*64*–*66*). In recent years, bioarchaeologists have also sought to apply this framework to studies of stress, frailty, and mortality in past populations, highlighting the relationship between certain skeletal indicators of physiological stress and early mortality [for detailed reviews of DOHaD in bioarchaeology, see (*67*–*69*)]. LEH, grooves on the teeth produced when the enamel formation process is interrupted (*70*, *71*), are particularly useful skeletal indicators of early life adversity as permanent teeth develop during childhood. Numerous bioarchaeological studies have reported increased mortality risks, earlier ages at death, and reduced survivorship among adults with LEH compared to those without LEH [e.g., (*9*, *11*, *48*, *72*, *73*)]. Like previous studies, the results of this study suggest that LEH presence is associated with significantly higher hazards of death, significantly higher risks of death, and significantly lower survivorship in medieval London, and LEH presence/absence is a primary contributor in skeletal indices of mortality and resilience.

Research in living populations indicates that individuals who experienced adverse events during growth and development, such as war or famine, often exhibited diminished height in adulthood, which is in turn associated with increased risks of mortality (*74*–*76*). Similarly, long bone lengths and stature estimates have been used in bioarchaeological studies to examine the relationship between physiological stress in early life and health outcomes in adulthood [e.g., (*46*, *77*, *78*)]. In this study, Kaplan-Meier survival analysis and Cox proportional hazards analysis demonstrated that short femoral length was associated with significantly lower survivorship and higher hazards of death, respectively. Likewise, the Spearman’s correlation indicated that femoral length significantly contributed to the revised mortality and resilience indices although to a lesser extent than other biomarkers, such as LEH. Consequently, this study suggests that adverse conditions during development capable of permanently restricting femoral growth, such as chronic malnutrition or infection, are significantly associated with measures of frailty, survivorship, and mortality.

### Morbidity and mortality in medieval London

Our comparison of frailty and resilience indices between males and females does not reveal any significant differences between estimated males and females. These findings suggest the male-female morbidity-mortality paradox that is observed in some present-day populations may not have existed in medieval London [this is consistent with DeWitte’s (*79*) findings using a single biomarker, dental caries]. It is possible that this lack of observable sex differences might indicate something important about the interaction of social and biological factors in medieval London. Numerous studies in human and nonhuman animal populations have produced evidence of lower age-specific mortality rates, lower severity of disease symptoms and disease-specific mortality rates, and longer life expectancies in females compared to males (*80*–*82*). These findings suggest greater physiological resilience or buffering in females compared to males. Some of these observed differences reflect variation in the production of gonadal hormones as estrogen generally enhances immune competence whereas testosterone can have an immune compromising effect [see (*80*)]. In contemporary human populations, exceptions to this general pattern are often explained by cultural practices that disproportionately benefit males and/or harm females, such as preferential access to nutritious foods and medical care for males (*82*). Our expectation given these findings from humans and other animals was to find sex differences that favor females. The lack of such a finding in this study may reflect cultural buffering that favored males in medieval England. Medieval England is often characterized as a patriarchal society, which might suggest that there was comparatively restricted access to health-promoting resources (e.g., abundant and nutritious diets) for females or greater exposure to factors that harmed female health. However, a distinction should be made between dominant ideologies of the time regarding the subjugation of women versus the actual experiences of women, of which there was variation based on factors like social class and economic conditions (*83*). Thus, future work integrating other lines of evidence reflective of things such as dietary quality may clarify if our findings are the result of sex-based or gender-based differences in cultural buffering.

The one aspect of our analysis that demonstrated differences between males and females was in the Spearman’s analyses (Table 6), specifically comparing short femur length with the 5-biomarker frailty (scalar) values. Among males, the correlation between shorter femoral length and 5-biomarker (scalar) frailty index value is not significant, whereas, among females, all correlations between femoral length and frailty and resilience index values are statistically significant. In addition, all correlations (rho values) between femoral lengths and index values are consistently higher in female than male samples. This discrepancy in the relative strength of the association between femur length (a proxy for stature) and frailty among adults may reflect the effects of selective mortality at younger ages. It might have been the case in medieval England that mortality during childhood and adolescence weeded out the frailest (i.e., shortest) males, and thus, they were not present in the adult sample used for this study. If such a selective effect was experienced by more males than females, perhaps due to biological buffering of the latter, then it may have resulted in adult cohorts in which stature was less variable among males than females, thereby resulting in weaker observed associations between stature and frailty for the males in this study. These results convey how not only the morbidity-mortality paradox but also the osteological paradox may be playing out in medieval London: With proportionately more males dying in childhood and adolescence, the surviving cohort of adult males—likely aided by cultural buffering—exhibits less variation in femur length compared with the variably frail adult females.

Other studies of the relationships among frailty, femur length, and mortality in medieval London have produced findings that conflict with those presented here. In an analysis of the East Smithfield cemetery, Hawks and colleagues (*84*) observed a pronounced disparity in femoral length among males who had experienced early life stressors (evinced by LEH) compared to males who had not experienced early life stressors. Although a disparity in femoral length was also observed among females with and without LEH, the discrepancy was not significant. Hawks and colleagues argue that this lack of a difference in femoral lengths among females who have and have not experienced early life stress may suggest a greater biological buffering capacity (or diminished reaction to stress events) among females compared to males in the medieval period. The authors also suggest that the result could be caused by selective mortality against females during childhood and adolescence, which would reduce the overall frailty of the surviving adult cohort of females (*84*). Similarly, an analysis of St. Mary Spital cemetery by DeWitte and Yaussy (*78*) found an association between femur length and death during famines among males but not females. Like Hawks and colleagues (*84*), DeWitte and Yaussy (*78*) suggest that the lack of an association between female femoral length and mortality during famines may be related to a higher buffering capacity among females or selective mortality among females during childhood and adolescence. The skeletal assemblages in both of the studies referenced above (i.e., East Smithfield and St. Mary Spital) are also included in the present study. Consequently, we suggest that the association between femur length and biological sex may be context-dependent. That is, the observed associations between short femora and biological sex may be more pronounced in particular burial contexts (e.g., East Smithfield) or during particular historical events (e.g., famines), and the relationship may be masked by the inclusion of other skeletal assemblages from medieval London in the present study.

### Current and future considerations in skeletal frailty

Later-life frailty reflects inherent, systemic, and detrimental alterations in human physiology and function secondary to surviving life’s stressors. Over the course of human evolution and historically, individuals have responded adaptively as physical and sociocultural stressors threatened their somatic stability and survival. Then and today, multiple environmental, physical, and social factors constrain individual physiology, function, health, and adaptability while modulating individual stress experiences, allostatic responsiveness, and later-life frailty. Assessing frailty among the living has improved current understandings of health and well-being among older members of our populations, while early detection, physical therapy, and medical interventions have made frailty a partly reversible phenotype. Both frailty and physiological dysregulation reflect innate abilities to return to a homeostatic equilibrium following stressor exposures although not at the same setpoints existing prior to stressor responses. At the same time, increasing frailty with age reflects individual resilience to life’s detrimental processes, debilitation, and mortality compared to those not surviving.

Biological and physiological anthropology, human biology, clinical medicine, and bioarchaeology share common goals, understanding how social, cultural, environmental, and biological factors interactively influence human health, wellness, and life span. Frailty estimates among the living and the deceased provide one avenue for examining and comparing these associations in both the present and the past. Integrating hazards and survival analyses with cumulative phenotype approaches, we created frailty and resilience indices specific to medieval London, drawing from theoretical concepts of local biologies (*61*, *62*). These population-specific frailty and resilience indices demonstrate more robust results than the original SFI, with results consistently reflecting decreased survivorship/increased hazard with higher frailty values and increased survivorship/decreased hazard with higher resilience values. In addition, correlations between individual biomarkers and frailty and resilience index values demonstrated how some lifetime stressors—e.g., LEH and PNBF—highly correlate with cumulative frailty, whereas others—e.g., stunted femoral length—minimally affect an individual’s lifetime frailty. When applied to estimated sex-based differences in a medieval London sample, both frailty and resilience values show no significant differences between males and females. These results demonstrate how a population-tailored frailty index may effectively capture risk of mortality and cumulative lifetime stress in this urban center.

## MATERIALS AND METHODS

### Cemeteries

The individuals analyzed in this study come from four burial grounds in London, all of which have been dated to the medieval period in England: East Smithfield, Guildhall Yard, St. Mary Graces, and St. Mary Spital (Table 9). Most of the metric and paleopathological data on the skeletal samples used in this study are derived from the Museum of London’s Centre for Human Bioarchaeology Wellcome Osteological Research Database (WORD). Pathological conditions were recorded and scored according to the Human Osteology Method Statement and standards therein (*85*). The exceptions are data on LEH and PNBF, which, in addition to sex and age estimates, are drawn from S.N.D.’s previous research on these sites. All individuals included in our analyses were those well preserved enough to provide sufficient data to estimate age and sex (see specific numbers for each analysis in the tables).

**Table 9.** Chronological and demographic data for original medieval samples. Data for East Smithfield, Guildhall Yard, and St. Mary Grace come from the WORD, and St. Mary Spital data come from S.N.D. (hence slightly different age groups). Percentages (%) are based on total individuals in cemetery sample, overall or by sex. *M*, estimated male/probable male; *F*, estimated female/probable female; *I*, estimated intermediate/indeterminate.

	Estimated age groups	All	%	*M*	%	*F*	%	*I*	%
East Smithfield	18–25 years	63	15	36	19	16	15.4	11	8.7
	26–35 years	121	28.8	68	36	38	36.5	15	11.8
	36–45 years	97	23.1	62	32.8	31	29.8	4	3.1
	>46 years	22	5.2	12	6.3	9	8.7	1	0.079
	Unclassified adult	117	27.9	11	5.8	10	9.6	96	75.6
	Total	420		189		104		127	
Guildhall Yard	18–25 years	7	14.9	5	27.8	2	13.3	0	0
	26–35 years	10	21.3	4	22.2	5	33.3	1	7.1
	36–45 years	11	23.4	7	38.9	3	20	1	7.1
	>46 years	6	12.8	1	5.6	5	33.3	0	0
	Unclassified adult	0	0	1	5.6	0	0	12	85.7
	Total	47		18		15		14	
St. Mary Grace	18–25 years	32	11.3	16	11.8	13	19.1	3	3.8
	26–35 years	58	20.5	31	22.8	23	33.8	4	5.1
	36–45 years	69	24.4	47	34.6	18	26.5	4	5.1
	>46 years	40	14.1	28	20.6	10	14.7	2	2.5
	Unclassified adult	84	29.7	14	10.3	4	5.9	66	83.5
	Total	283		136		68		79	
St. Mary Spital	18–25.99 years	268	29.5	77	20.5	147	30.7	44	77.2
	26–35.99 years	328	36.1	168	44.8	160	33.4	0	0
	36–45.99 years	152	16.7	68	18.1	84	17.5	0	0
	>46 years	144	15.9	59	15.7	82	17.1	3	5.3
	Unclassified adult	16	1.8	3	0.8	6	1.3	10	17.5
	Total	908		375		479		57	

#### East Smithfield cemetery

Before excavation, the East Smithfield cemetery was located in northeast London, near the Tower of London. The cemetery is unique in that it is one of the few excavated cemeteries in England with archaeological and documentary evidence linking it to the first wave of the Second Pandemic of Plague (now often referred to as the Black Death), which affected London in the mid-14th century (*86*). Records from the Church of the Holy Trinity note the location of the cemetery and its dimensions, as well as the fact that the cemetery was founded in 1348 with the sole purpose of interring victims of the Black Death (*85*). Archaeological evidence further indicates that interments were completed in a single phase and there is no evidence to indicate any individuals were interred after Black Death mortality subsided in 1350 (*86*).

#### Guildhall Yard

Guildhall Yard was originally the site of the lay cemetery of St. Lawrence Jewry in central London. Between 1992 and 1997, the Museum of London Archaeology Service excavated a total of 68 individuals, most of whom were interred during the 11th and 12th centuries (*87*).

#### St. Mary Graces cemetery

Located in the same area of London as the East Smithfield cemetery, the St. Mary Graces cemetery was associated with the Cistercian Abbey of St. Mary Graces, which was established in 1350 shortly after the Black Death, and the cemetery continued to be used throughout the medieval period until the Reformation in 1538 (*88*). The cemetery consists of members of the general population, whereas important laypeople and the monks of the Abbey were interred in the church and chapels of the Abbey (*88*, *89*).

#### St. Mary Spital cemetery

The St. Mary Spital cemetery was associated with the medieval priory and hospital of St. Mary Spital, which was founded in 1197 and located just outside the city of London. The hospital was intended to serve the impoverished inhabitants of the city, as well as pilgrims, travelers, and women in childbirth, but the cemetery is also believed to draw from communities in London and the surrounding areas (*90*). Burials from the St. Mary Spital cemetery were divided into four phases based on radiometric dating of the site’s stratigraphy: period 14 (1120 to 1200 CE), period 15 (1200 to 1250 CE), period 16 (1250 to 1400 CE), and period 17 (1400 to 1539 CE) (*91*, *92*). The burials in the St. Mary Spital cemetery have been also divided into four types, based on the number and arrangement of individuals in the grave cut. Type A burials consist of single interments, whereas type B burials consist of small groups of bodies distributed in a horizontal layer, and type C burials consist of small groups of bodies distributed vertically within the grave cut (*90*, *92*). Type D burials, in contrast, consist of multiple horizontal layers of bodies stacked on top of each other in a single grave cut, and the manner in which these burials were dug suggests that they were constructed to accommodate a large number of bodies within a short period of time. As a result, the type D burials are known as “the catastrophic group,” and evidence suggests that they may be associated with famines detailed in historical texts (*93*).

### Methods

#### Age and sex estimation

Adult ages were estimated by S.N.D. using transition analysis (*94*). Transition analysis is a Bayesian method of age estimation designed to avoid the major biases associated with traditional methods, including age mimicry of the known-age reference sample and the use of a broad terminal age group for the oldest adults in a sample (which typically lumps together all adults estimated to be 45 years of age or older). In this study, transition analysis was used to generate age at death point estimates for each individual, as well as SEs associated with those age estimates (only the point estimates were used in the analyses for this study).

Phenotypic sex was estimated from a combination of morphoscopic traits of the os coxa and cranial morphology (*95*). Only estimated females and males were included in the analysis, so biological variables associated with phenotypic sex could be considered in the analysis. For example, when assessing stunting in individuals, differences in size between the sexes should be considered as females, on average, tend to be shorter than male counterparts. Such criteria for evaluating stunting in intermediate individuals do not currently exist, hence their regrettable exclusion.

#### Skeletal frailty indices

SFIs were constructed and tested for this sample from six and five biomarkers, respectively (table S4): LEH, PD, OA, PNBF, trauma, and stunted (2 SDs below sex-specific average) femoral length; and LEH, PD, OA, PNBF, and trauma. These biomarkers were selected as robust proxies for lifetime stress, capturing both childhood (stunted femoral length and LEH) and adulthood (PD, OA, PNBF, and trauma) episodes of acute and chronic stress (*13*, *56*, *59*). LEH and PNBF were scored for the presence and number and activity, respectively, by S.N.D. [see (*53*, *96*)], whereas PD, OA, trauma, and femoral length data were obtained from WORD (*87*). Where applicable, individual biomarkers were scored based on the presence/absence and severity/lesion activity (i.e., active lesions were defined as woven/unremodeled, and healed lesions were defined as sclerotic/remodeled; severity was defined by the progression of a condition, such as the progressive alveolar resorption of PD), which resulted in four skeletal frailty indices: 5-biomarker and 6-biomarker indices using binary (presence/absence) data and 5-biomarker and 6-biomarker indices using scalar (severity/lesion activity) data. For example, an active PNBF lesion would receive a binary score of “1” and scalar score of “1,” but a healed PNBF lesion would receive a binary score of “1” and scalar score of “0.33.” However, with OA and femoral length, only binary high “1” and low “0” frailty scores were used. For OA, frailty was based on the presence or absence of the condition; for femoral length, we considered high frailty to be indicated when an individual’s measurement was 2 SDs below the male or female average. Criteria for ascribed “high” and “low” frailty scores for each biomarker are outlined in table S5.

As previous research on medieval London skeletal assemblages has shown (*12*), SFIs with fewer biomarkers increase the number of individuals that can be included in analyses without sacrificing indexical informativeness. However, as preservation oftentimes limits measurements, and consequently sample size, for the 5-biomarker SFI, we removed femoral length as a variable to increase sample size but retained LEH as an indicator of survived childhood stress.

#### Survival and hazards analysis

One potential concern with frailty indices, as they have been designed and used to date in bioarchaeology, is that they do not fully account for the possibility that all skeletal biomarkers may not equally reflect the same information about frailty or resilience. For example, OA tends to be found at higher frequencies in older adults and may thus most appropriately be viewed as a marker of resilience, whereas numerous studies have found higher risks of early death associated with LEH, which thus may be a strong indicator of frailty. With this in mind, we applied survival and hazards analysis in three stages to (1) assess what the original SFIs may reflect regarding frailty or resilience overall, (2) determine the individual contribution of each skeletal biomarker to the results observed using the original SFIs and how those markers might be more appropriately combined in revised indices (frailty and resilience), and (3) examine how the revised indices perform.

The associations between survivorship and the (1) original indices, (2) individual biomarkers, and (3) revised indices were assessed using nonparametric Kaplan-Meier survival analysis. Note that the sample sizes vary by analysis; analysis of the original and revised indices (steps 1 and 3) are limited to individuals who could be scored for all relevant biomarkers (which inherently reduces sample sizes), whereas analysis of each of the individual biomarkers (step 2) used a sample that included all individuals scored for that specific biomarker. We use a log rank test to identify significant differences in survivorship based on index values and, for the individual biomarkers, the presence and/or scalar value of each biomarker. All Kaplan-Meier survival analyses were conducted in SPSS version 29.

Associations between hazards of death and indices/biomarkers were analyzed using the semiparametric Cox proportional hazards model and the fully parametric Gompertz model of adult mortality. The Cox model does not require estimation of the parameters of the baseline hazard function and tests the null hypothesis that the covariate (in this case, the presence of one or more biomarkers) has no effect on the hazard, with the reported hazard ratio indicating the change in risk of death associated with a unit increase in the covariate. For the Cox analyses for each biomarker, we specified absence as the reference value for comparison; for analyses of the indices, we specified an SFI of 0 (i.e., no observable biomarkers) as the reference value. The same data were used for Cox analyses regardless of whether we specified a reference. The advantage of specifying a reference value is that it allows us to identify variation in the hazards of death (relative to the reference value) across all values of the index greater than the reference value. If the reference value is not specified, then the results of Cox analyses reveal how the hazards of death change with each unit increase in the covariate, but those results are difficult to interpret if they are (i) nonsignificant and (ii) there is not a consistent pattern of increased or decreased hazards of death associated with unit increases in the covariate. We used age at death (with birth, i.e., age 0, as the baseline) as the timescale variable for each Cox analysis and the outcome is the hazard ratio, indicating whether hazards of death increased or decreased with changes in the covariate value. When considering which individual biomarkers to include in the revised indices, we assessed the survival functions to evaluate potential violations of the proportionality assumption of the Cox model; only those biomarkers for which no crossing-over of survival functions existed were included in the revised indices. All Cox analyses were conducted in SPSS version 29.

The Gompertz model has two parameters (*97*): one specifying the relatively low risk of mortality typical of younger adult ages and the other specifying the rate at which the risk increases with senescence (*98*). For this study, biomarker presence or index value was modeled as a covariate affecting the Gompertz model using a proportional hazard specification

$$h_{i}\left(t_{i}|x_{i}\rho \right)=h\left(t_{i}\right)e^{\left(x_{i}\rho \right)}$$

where the baseline Gompertz hazard *h*(*t_i_*) *=* α*e*^β*t*^, *t_i_* is the age of the *i*th skeleton in years, *x_i_* is the biomarker covariate, and ρ is the parameter representing the effect of the covariate on the baseline hazard. We estimated the Gompertz model parameters with Holman’s program mle (*99*). A likelihood ratio test (LRT) was used to assess the fit of the full model (i.e., the Gompertz model in which all parameters—including the biomarker/indices covariate—are estimated) compared to the baseline model (i.e., the Gompertz model in which the covariate is set to zero). For individual biomarkers, the LRT therefore tests the null hypothesis that there is no difference in risk of mortality between people with and without biomarkers, and for the indices, it tests the null hypothesis that increasing index values has no effect on risk of death (*H*_0_: effect of biomarker/index covariate = 0). The LRT was computed in Excel as follows: LRT = −2[ln(*L*_biomarker/index_) − ln(*L*_baseline_)], where LRT approximates a χ^2^ distribution with *df* = 1. For all analyses, we selected a priori an alpha level of 0.1 as indicative of a trend. All data from the original 5-biomarker and 6-biomarker SFI and new 4-biomarker and 5-biomarker frailty and resilience indices are available in data S1.

#### Evaluating frailty and resilience scores with Spearman’s correlations and logistic regression

Frailty and resilience indices were compared with individual biomarker scores through Spearman’s correlations to assess the relationship between specific biomarkers of frailty/resilience and total frailty/resilience. The strength of this correlation intimates what variables are affecting frailty/resilience in this population and is important for understanding childhood and adulthood stress affecting frailty and resilience (i.e., DOHaD) as well as observing possible variability in frailty and resilience biomarkers between females and males (e.g., morbidity-mortality paradox).

Frailty and resilience index values between males and females were assessed through binary logistic regression. Estimated sex was set as the dependent variable and estimated age and index values as covariates. All assumptions for logistic regression were met to conduct this test of the specified data. Results are viewable in Table 8 for 4-biomarker and 5-biomarker binary and scalar frailty indices and 4-biomarker binary and scalar resilience indices. All statistical tests comparing frailty and resilience scores (i.e., Spearman’s correlations and logistic regression) were conducted in SPSS version 29.

## References

[1] L. P. Fried, C. M. Tangen, J. Walston, A. B. Newman, C. Hirsch, J. Gottdiener, T. Seeman, R. Tracy, W. J. Kop, G. Burke, M. A. McBurnie, Frailty in older adults: Evidence for a phenotype. J. Gerontol. A Biol. Sci. Med. Sci. 56, M146–M157 (2001). 11253156 10.1093/gerona/56.3.m146

[2] D. E. Crews, Aging, frailty, and design of built environments. J. Physiol. Anthropol. 41, 2 (2022). 34980249 10.1186/s40101-021-00274-w PMC8725353

[3] J. Walston, E. C. Hadley, L. Ferrucci, J. M. Guralnik, A. B. Newman, S. A. Studenski, W. B. Ershler, T. Harris, L. P. Fried, Research agenda for frailty in older adults: Toward a better understanding of physiology and etiology: Summary from the American geriatrics society/national institute on aging research conference on frailty in older adults. J. Am. Geriatr. Soc. 54, 991–1001 (2006). 16776798 10.1111/j.1532-5415.2006.00745.x

[4] B. J. Cohen, S. A. Jones, *Medical Terminology: An Illustrated Guide* (Jones & Bartlett Learning, ed. 9, 2021).

[5] N. Cross, D. McWay, *Stanfield’s Essential Medical Terminology* (Jones & Bartlett Learning, ed. 5, 2020).

[6] J. W. Wood, G. R. Milner, H. C. Harpending, K. M. Weiss, The osteological paradox: Problems of inferring prehistoric health from skeletal samples. Curr. Anthropol. 33, 343–370 (1992).

[7] J. W. Vaupel, K. G. Manton, E. Stallard, The impact of heterogeneity in individual frailty on the dynamics of mortality. Demography 16, 439–454 (1979). 510638

[8] J. J. Wilson, Paradox and promise: Research on the role of recent advances in paleodemography and paleoepidemiology to the study of “health” in Precolumbian societies. Am. J. Phys. Anthropol. 155, 268–280 (2014). 25146753 10.1002/ajpa.22601

[9] S. N. DeWitte, J. W. Wood, Selectivity of Black Death mortality with respect to preexisting health. Proc. Natl. Acad. Sci. U.S.A. 105, 1436–1441 (2008). 18227518 10.1073/pnas.0705460105 PMC2234162

[10] K. Godde, V. Pasillas, A. Sanchez, Survival analysis of the Black Death: Social inequality of women and the perils of life and death in Medieval London. Am. J. Phys. Anthropol. 173, 168–178 (2020). 32472637 10.1002/ajpa.24081

[11] S. L. Yaussy, S. N. DeWitte, R. C. Redfern, Frailty and famine: Patterns of mortality and physiological stress among victims of famine in medieval London. Am. J. Phys. Anthropol. 160, 272–283 (2016). 26854255 10.1002/ajpa.22954

[12] K. E. Marklein, D. E. Crews, Frail or hale: Skeletal frailty indices in Medieval London skeletons. PLOS ONE 12, e0176025 (2017). 28467438 10.1371/journal.pone.0176025 PMC5415061

[13] K. E. Marklein, R. E. Leahy, D. E. Crews, In sickness and in death: Assessing frailty in human skeletal remains. Am. J. Phys. Anthropol. 161, 208–225 (2016). 27312014 10.1002/ajpa.23019

[14] D. E. Crews, K. E. Marklein, “Conceptualizing frailty in the quick and the dead” in *Anthropological Perspectives on Aging* (University Press of Florida, 2023), pp. 249–270.

[15] S. N. DeWitte, S. L. Yaussy, Sex differences in adult famine mortality in medieval London. Am. J. Phys. Anthropol. 171, 164–169 (2020). 31587269 10.1002/ajpa.23930

[16] B. Wyatt, C. McFadden, S. Ward, L. A. B. Wilson, Assessing the association of skeletal indicators of stress with mean age-at-death in sub-adults. Am. J. Biol. Anthropol. 182, 440–451 (2023). 37610235 10.1002/ajpa.24833

[17] K. E. Marklein, D. E. Crews, Highs and lows of frailty: Skeletal frailty differentials among socioeconomic groups in Postmedieval London. Archaeol. Anthropol. Sci. 14, 43 (2022).

[18] L. P. Fried, A. A. Cohen, Q.-L. Xue, J. Walston, K. Bandeen-Roche, R. Varadhan, The physical frailty syndrome as a transition from homeostatic symphony to cacophony. Nat. Aging 1, 36–46 (2021). 34476409 10.1038/s43587-020-00017-z PMC8409463

[19] A. A. Sayer, A. Cruz-Jentoft, Sarcopenia definition, diagnosis and treatment: Consensus is growing. Age Ageing 51, afac220 (2022). 36273495 10.1093/ageing/afac220 PMC9588427

[20] X. Song, A. Mitnitski, K. Rockwood, Prevalence and 10-year outcomes of frailty in older adults in relation to deficit accumulation. J. Am. Geriatr. Soc. 58, 681–687 (2010). 20345864 10.1111/j.1532-5415.2010.02764.x

[21] E. Dent, C. Lien, W. S. Lim, W. C. Wong, C. H. Wong, T. P. Ng, J. Woo, B. Dong, S. de la Vega, P. J. H. Poi, The Asia-Pacific clinical practice guidelines for the management of frailty. J. Am. Med. Dir. Assoc. 18, 564–575 (2017). 28648901 10.1016/j.jamda.2017.04.018

[22] K. Bandeen-Roche, C. L. Seplaki, J. Huang, B. Buta, R. R. Kalyani, R. Varadhan, Q.-L. Xue, J. D. Walston, J. D. Kasper, Frailty in older adults: A nationally representative profile in the United States. J. Gerontol. A Biol. Sci. Med. Sci. 70, 1427–1434 (2015). 26297656 10.1093/gerona/glv133 PMC4723664

[23] K. Rockwood, X. Song, C. MacKnight, H. Bergman, D. B. Hogan, I. McDowell, A. Mitnitski, A global clinical measure of fitness and frailty in elderly people. CMAJ 173, 489–495 (2005). 16129869 10.1503/cmaj.050051 PMC1188185

[24] S. Studenski, R. P. Hayes, R. Q. Leibowitz, R. Bode, L. Lavery, J. Walston, P. Duncan, S. Perera, Clinical global impression of change in physical frailty: Development of a measure based on clinical judgment. J. Am. Geriatr. Soc. 52, 1560–1566 (2004). 15341562 10.1111/j.1532-5415.2004.52423.x

[25] J. De Lepeleire, A. W. Wind, S. Iliffe, E. D. Moniz-Cook, J. Wilcock, V. M. González, E. Derksen, M. V. Gianelli, M. Vernooij-Dassen, The primary care diagnosis of dementia in Europe: An analysis using multidisciplinary, multinational expert groups. Aging Ment. Health 12, 568–576 (2008). 18855172 10.1080/13607860802343043

[26] J.-M. Robine, J.-P. Michel, F. R. Herrmann, Who will care for the oldest people in our ageing society? BMJ 334, 570–571 (2007). 17363829 10.1136/bmj.39129.397373.BE PMC1828348

[27] L. Rodríguez-Mañas, C. Féart, G. Mann, J. Viña, S. Chatterji, W. Chodzko-Zajko, M. Gonzalez-Colaço Harmand, H. Bergman, L. Carcaillon, C. Nicholson, Searching for an operational definition of frailty: A Delphi method based consensus statement. The frailty operative definition-consensus conference project. J. Gerontol. A Biol. Sci. Med. Sci. 68, 62–67 (2013). 22511289 10.1093/gerona/gls119 PMC3598366

[28] T. L. Gruenewald, T. E. Seeman, A. S. Karlamangla, C. A. Sarkisian, Allostatic load and frailty in older adults. J. Am. Geriatr. Soc. 57, 1525–1531 (2009). 19682116 10.1111/j.1532-5415.2009.02389.x PMC3650612

[29] S. L. Szanton, J. K. Allen, C. L. Seplaki, K. Bandeen-Roche, L. P. Fried, Allostatic load and frailty in the women’s health and aging studies. Biol. Res. Nurs. 10, 248–256 (2009). 18829589 10.1177/1099800408323452 PMC2730583

[30] A. Ghosh, M. Kundu, N. Devasenapathy, M. Woodward, V. Jha, Frailty among middle-aged and older women and men in India: Findings from wave 1 of the longitudinal Ageing study in India. BMJ Open 13, e071842 (2023). 10.1136/bmjopen-2023-071842 PMC10391831 37524559

[31] Y. Lu, X. Gwee, D. Q. Chua, C. T. Tan, K. B. Yap, A. Larbi, T. P. Ng, Physiological dysregulation, frailty, and impacts on adverse health and functional outcomes. Front. Med. 8, 751022 (2021). 10.3389/fmed.2021.751022 PMC8569246 34746185

[32] C. McCrory, S. McLoughlin, R. Layte, C. NiCheallaigh, A. M. O’Halloran, H. Barros, L. F. Berkman, M. Bochud, E. M. Crimmins, M. T. Farrell, Towards a consensus definition of allostatic load: A multi-cohort, multi-system, multi-biomarker individual participant data (IPD) meta-analysis. Psychoneuroendocrinology 153, 106117 (2023). 37100008 10.1016/j.psyneuen.2023.106117 PMC10620736

[33] G. R. Milner, J. L. Boldsen, Life not death: Epidemiology from skeletons. Int. J. Paleopathol. 17, 26–39 (2017). 28521910 10.1016/j.ijpp.2017.03.007

[34] T. Waldron, *Paleoepidemiology: The Measure of Disease in the Human Past*, University College London Institute of Archaeology Publications (Left Coast Press Inc., 2007).

[35] D. A. Weston, Investigating the specificity of periosteal reactions in pathology museum specimens. Am. J. Phys. Anthropol. 137, 48–59 (2008). 18398845 10.1002/ajpa.20839

[36] N. E. Smith-Guzmán, The skeletal manifestation of malaria: An epidemiological approach using documented skeletal collections. Am. J. Phys. Anthropol. 158, 624–635 (2015). 26213353 10.1002/ajpa.22819

[37] D. Dangvard Pedersen, G. R. Milner, H. J. Kolmos, J. L. Boldsen, The association between skeletal lesions and tuberculosis diagnosis using a probabilistic approach. Int. J. Paleopathol. 27, 88–100 (2019). 30661884 10.1016/j.ijpp.2019.01.001

[38] L. O’Donnell, J. E. Buikstra, E. C. Hill, A. S. Anderson, M. J. O’Donnell Jr., Skeletal manifestations of disease experience: Length of illness and porous cranial lesion formation in a contemporary juvenile mortality sample. Am. J. Hum. Biol. 35, e23896 (2023). 36974669 10.1002/ajhb.23896

[39] F. A. Crespo, C. K. Klaes, A. E. Switala, S. N. DeWitte, Do leprosy and tuberculosis generate a systemic inflammatory shift? Setting the ground for a new dialogue between experimental immunology and bioarchaeology. Am. J. Phys. Anthropol. 162, 143–156 (2017). 27704524 10.1002/ajpa.23104

[40] J. W. Vaupel, Inherited frailty and longevity. Demography 25, 277–287 (1988). 3396752

[41] K. van Schaik, D. Vinichenko, F. Rühli, Health is not always written in bone: Using a modern comorbidity index to assess disease load in paleopathology. Am. J. Phys. Anthropol. 154, 215–221 (2014). 24936606 10.1002/ajpa.22494

[42] S. M. Hens, K. Godde, K. M. Macak, Iron deficiency anemia, population health and frailty in a modern Portuguese skeletal sample. PLOS ONE 14, e0213369 (2019). 30845224 10.1371/journal.pone.0213369 PMC6405098

[43] L. O’Donnell, E. Moes, Sex differences in linear enamel hypoplasia prevalence and frailty in Ancestral Puebloans. J. Archaeol. Sci. Rep. 39, 103153 (2021).

[44] R. Watts, The long-term impact of developmental stress. Evidence from later medieval and post-medieval London (AD1117–1853). Am. J. Phys. Anthropol. 158, 569–580 (2015). 26172272 10.1002/ajpa.22810

[45] S. L. Yaussy, S. N. DeWitte, Calculus and survivorship in medieval London: The association between dental disease and a demographic measure of general health. Am. J. Phys. Anthropol. 168, 552–565 (2019). 30613949 10.1002/ajpa.23772

[46] S. N. DeWitte, G. Hughes-Morey, Stature and frailty during the Black Death: The effect of stature on risks of epidemic mortality in London, A.D. 1348-1350. J. Archaeol. Sci. 39, 1412–1419 (2012). 10.1016/j.jas.2012.01.019 PMC3868458 24363485

[47] K. S. Kelmelis, M. H. Price, J. Wood, The effect of leprotic infection on the risk of death in medieval rural Denmark. Am. J. Phys. Anthropol. 164, 763–775 (2017). 28940226 10.1002/ajpa.23314

[48] A. C. Ham, D. H. Temple, H. D. Klaus, D. R. Hunt, Evaluating life history trade-offs through the presence of linear enamel hypoplasia at Pueblo Bonito and Hawikku: A biocultural study of early life stress and survival in the Ancestral Pueblo Southwest. Am. J. Hum. Biol. 33, e23506 (2021). 32924230 10.1002/ajhb.23506

[49] A. Wissler, S. N. DeWitte, Frailty and survival in the 1918 influenza pandemic. Proc. Natl. Acad. Sci. U.S.A. 120, e2304545120 (2023). 37812724 10.1073/pnas.2304545120 PMC10589609

[50] S. N. DeWitte, G. Hughes-Morey, J. Bekvalac, J. Karsten, Wealth, health and frailty in industrial-era London. Ann. Hum. Biol. 43, 241–254 (2016). 26073638 10.3109/03014460.2015.1020873

[51] C. McFadden, M. F. Oxenham, A paleoepidemiological approach to the osteological paradox: Investigating stress, frailty and resilience through cribra orbitalia. Am. J. Phys. Anthropol. 173, 205–217 (2020). 32578874 10.1002/ajpa.24091

[52] L. O’Donnell, Indicators of stress and their association with frailty in the precontact Southwestern United States. Am. J. Phys. Anthropol. 170, 404–417 (2019). 31294832 10.1002/ajpa.23902

[53] S. N. DeWitte, Differential survival among individuals with active and healed periosteal new bone formation. Int. J. Paleopathol. 7, 38–44 (2014). 29539489 10.1016/j.ijpp.2014.06.001

[54] D. J. Kim, M. S. Massa, C. M. Potter, R. Clarke, D. A. Bennett, Systematic review of the utility of the frailty index and frailty phenotype to predict all-cause mortality in older people. Syst. Rev. 11, 187 (2022). 36056441 10.1186/s13643-022-02052-w PMC9438224

[55] R. H. Steckel, J. C. Rose, *The Backbone of History: Health and Nutrition in the Western Hemisphere* (Cambridge Univ. Press, 2002).

[56] R. H. Steckel, C. S. Larsen, P. W. Sciulli, P. L. Walker, *The Global History of Health Project. Data Collection Codebook* (The Ohio State University, 2005).

[57] M. Hubbe, M. K. Green, C. M. Cheverko, W. A. Neves, Brief communication: A re-evaluation of the health index of southern Brazilian shellmound populations. Am. J. Phys. Anthropol. 165, 353–362 (2018). 29090738 10.1002/ajpa.23346

[58] A. C. Tuggle, K. E. Marklein, D. E. Crews, Skeletal frailty at kałdus, a medieval Poland early Piast dynasty cemetery. Coll. Antropol. 45, 11–23 (2021).

[59] N. Zedda, B. Bramanti, E. Gualdi-Russo, E. Ceraico, N. Rinaldo, The biological index of frailty: A new index for the assessment of frailty in human skeletal remains. Am. J. Phys. Anthropol. 176, 459–473 (2021). 34418072 10.1002/ajpa.24394

[60] P. Stuart-Macadam, “Anaemia in Roman Britain: Poundbury Camp” in *Health in Past Societies: Biocultural Interpretations of Human Skeletal Remains in Archaeological Contexts* (BAR Publishing, 1991), vol. 567, pp. 101–113.

[61] M. Lock, Recovering the body. Ann. Rev. Anthropol. 46, 1–14 (2017).

[62] P. C. Kontos, Local biology: Bodies of difference in ageing studies. Ageing. Soc. 19, 677–689 (1999).

[63] D. J. Reid, M. C. Dean, Variation in modern human enamel formation times. J. Hum. Evol. 50, 329–346 (2006). 16300817 10.1016/j.jhevol.2005.09.003

[64] D. J. Barker, The fetal and infant origins of adult disease. BMJ 301, 1111 (1990). 2252919 10.1136/bmj.301.6761.1111 PMC1664286

[65] D. J. Barker, K. M. Godfrey, P. D. Gluckman, J. E. Harding, J. A. Owens, J. S. Robinson, Fetal nutrition and cardiovascular disease in adult life. Lancet 341, 938–941 (1993). 8096277 10.1016/0140-6736(93)91224-a

[66] P. D. Gluckman, M. A. Hanson, “The developmental origins of health and disease” in *Early Life Origins of Health and Disease* (Springer, 2006), pp. 1–7.

[67] S. C. Agarwal, P. Beauchesne, “It is not carved in bone: Development and plasticity of the aged skeleton” in *Social Bioarchaeology* (Wiley-Blackwell Publishing, 2011), pp. 312–332.

[68] R. L. Gowland, Entangled lives: Implications of the developmental origins of health and disease hypothesis for bioarchaeology and the life course. Am. J. Phys. Anthropol. 158, 530–540 (2015). 26767348 10.1002/ajpa.22820

[69] D. H. Temple, Bioarchaeological evidence for adaptive plasticity and constraint: Exploring life-history trade-offs in the human past. Evol. Anthropol. 28, 34–46 (2019). 30561095 10.1002/evan.21754

[70] A. H. Goodman, G. J. Armelagos, Factors affecting the distribution of enamel hypoplasias within the human permanent dentition. Am. J. Phys. Anthropol. 68, 479–493 (1985). 3909823 10.1002/ajpa.1330680404

[71] A. H. Goodman, J. C. Rose, Assessment of systemic physiological perturbations from dental enamel hypoplasias and associated histological structures. Am. J. Phys. Anthropol. 33, 59–110 (1990).

[72] G. J. Armelagos, A. H. Goodman, K. N. Harper, M. L. Blakey, Enamel hypoplasia and early mortality: Bioarcheological support for the Barker hypothesis. Evol. Anthropol. 18, 261–271 (2009).

[73] A. H. Goodman, G. J. Armelagos, Childhood stress and decreased longevity in a prehistoric population. Am. Anthropol. 90, 936–944 (1988).

[74] V. J. Felitti, R. F. Anda, D. Nordenberg, D. F. Williamson, A. M. Spitz, V. Edwards, J. S. Marks, Relationship of childhood abuse and household dysfunction to many of the leading causes of death in adults: The Adverse Childhood Experiences (ACE) Study. Am. J. Prev. Med. 14, 245–258 (1998). 9635069 10.1016/s0749-3797(98)00017-8

[75] N. C. Rodney, C. J. Mulligan, A biocultural study of the effects of maternal stress on mother and newborn health in the Democratic Republic of Congo. Am. J. Phys. Anthropol. 155, 200–209 (2014). 25043696 10.1002/ajpa.22568

[76] Z. Thayer, C. Barbosa-Leiker, M. McDonell, L. Nelson, D. Buchwald, S. Manson, Early life trauma, post-traumatic stress disorder, and allostatic load in a sample of American Indian adults. Am. J. Hum. Biol. 29, e22943 (2017). 10.1002/ajhb.22943 PMC5432430 27901290

[77] J. Geber, E. Murphy, Scurvy in the Great Irish Famine: Evidence of vitamin C deficiency from a mid-19th century skeletal population. Am. J. Phys. Anthropol. 148, 512–524 (2012). 22460661 10.1002/ajpa.22066 PMC3467765

[78] S. N. DeWitte, S. L. Yaussy, Femur length and famine mortality in medieval London. Bioarchaeol. Int. 1, 171–182 (2017).

[79] S. N. DeWitte, Assessing the existence of the male–female health-survival paradox in the past: Dental caries in medieval London. Am. J. Biol. Anthropol. 185, e24990 (2024). 38923302 10.1002/ajpa.24990

[80] S. L. Klein, The effects of hormones on sex differences in infection: From genes to behavior. Neurosci. Biobehav. Rev. 24, 627–638 (2000). 10940438 10.1016/s0149-7634(00)00027-0

[81] A. Oksuzyan, I. Petersen, H. Stovring, P. Bingley, J. W. Vaupel, K. Christensen, The male–female health–survival paradox: A survey and register study of the impact of sex-specific selection and information bias. Ann. Epidemiol. 19, 504–511 (2009). 19457685 10.1016/j.annepidem.2009.03.014 PMC2696561

[82] S. Stinson, Sex differences in environmental sensitivity during growth and development. Am. J. Phys. Anthropol. 28, 123–147 (1985).

[83] S. H. Rigby, Gendering the Black Death: Women in later medieval England. Gend. Hist. 12, 745–754 (2000).

[84] S. M. Hawks, K. Godde, S. M. Hens, The impact of early childhood stressors on later growth in medieval and postmedieval London. Int. J. Osteoarchaeol. 32, 804–812 (2022).

[85] N. Powers, *Human Osteology Method Statement* (Museum of London, 2008).

[86] I. Grainger, D. Hawkins, L. Cowal, R. Mikulski, *The Black Death Cemetery*, *East Smithfield*, *London Museum of London Archaeology Service Monograph 43* (Museum of London Archaeology Service, 2008).

[87] Museum of London, WORD Database, https://museumoflondon.org.uk/collections/other-collection-databases-and-libraries/centre-human-bioarchaeology/osteological-database/medieval.

[88] I. Grainger, D. Hawkins, Excavations at the Royal Mint site 1986-1988. Lond. Archaeol. 5, 429–436 (1988).

[89] I. Grainger, C. Phillpotts, *The Cistercian Abbey of St Mary Graces, East Smithfield, London. MoLA Monograph 44*, MOLA Monograph Series (Museum of London Archaeology, 2011).

[90] B. Connell, A. Gray Jones, R. Redfern, D. Walker, *A Bioarchaeological Study of Medieval Burials on the Site of St. Mary Spital: Excavations at Spitalfields Market*, *London E1:1991-2007* (Museum of London Archaeology, 2012).

[91] J. Sidell, C. Thomas, A. Bayliss, Validating and improving archaeological phasing at St. Mary Spital, London. Radiocarbon 49, 593–610 (2007).

[92] C. Thomas, B. Sloane, C. Phillpotts, *Excavations at the Priory and Hospital of St Mary Spital*, *London* (Museum of London Archaeology Service, 1997).

[93] A. Gray Jones, “Defining catastrophe: Mass burial at St Mary Spital” in *A Bioarchaeological Study of Medieval Burials on the Site of St. Mary Spital: Excavations at Spitalfields Market*, *London E1:1991-2007*, B. Connell, A. Gray Jones, R. Redfern, D. Walker, Eds. (Museum of London Archaeology, 2012), pp. 217–231.

[94] J. L. Boldsen, G. R. Milner, L. W. Konigsberg, J. W. Wood, “Transition analysis: A new method for estimating age from skeletons” in *Paleodemography: Age Distributions from Skeletal Samples*, R. D. Hoppa, J. W. Vaupel, Eds. (Cambridge Univ. Press, 2002), pp. 73–106.

[95] J. E. Buikstra, D. H. Ubelaker, *Standards for Data Collection from Human Skeletal Remains* (Arkansas Archaeological Society, 1994).

[96] S. N. DeWitte, Stress, sex, and plague: Patterns of developmental stress and survival in pre- and post-Black Death London. Am. J. Hum. Biol. 30, e23073 (2018). 10.1002/ajhb.23073 29071763

[97] B. Gompertz, On the nature of the function expressive of the law of human mortality, and on a new mode of determining the value of life contingencies. Philos. Trans. R. Soc. Lond. B Biol. Sci. 115, 513–583 (1825). 10.1098/rstb.2014.0379 PMC4360127 25750242

[98] J. W. Wood, D. J. Holman, K. A. O’Connor, R. J. Ferrell, “Mortality models for paleodemography” in *Paleodemography: Age Distributions from Skeletal Samples*, R. D. Hoppa, J. W. Vaupel, Eds. (Cambridge Univ. Press, 2002), pp. 129–168.

[99] D. J. Holman, mle: A programming language for building likelihood models (2005).

