# Supplementary material for: Frailty or resilience? Hazard-based and cumulative phenotype approaches to discerning signals of health inequality in medieval London
Source: Sci Adv. 2024 Nov 13;10(46):eadq5703. doi: 10.1126/sciadv.adq5703 (PMC11559611; doi:10.1126/sciadv.adq5703)
Supplement: Supplementary file 1 — Tables S1 to S5 Legend for data S1 [file sciadv.adq5703_sm.pdf]

Science Advances
AAAS

Supplementary Materials for

Frailty or resilience? Hazard-based and cumulative phenotype approaches to discerning signals of health inequality in medieval London

Samantha L. Yaussy *et al.*

Corresponding author: Samantha L. Yaussy, yaussysl@jmu.edu;
Kathryn E. Marklein, kathryn.marklein@louisville.edu

Sci. Adv. **10**, eadq5703 (2024)
DOI: 10.1126/sciadv.adq5703

The PDF file includes:

Tables S1 to S5
Legend for data S1

Other Supplementary Material for this manuscript includes the following:

Data S1

Table S1. Kaplan-Meier analyses of original 5- and 6-biomarker SFIs using presence/absence (binary) data. Mean survival times (mean ages at death) in years are shown with 95% confidence intervals in parentheses.

**Index**	**Index Score**	**Mean Age at Death (95% CI)**	***p***
**Original 5-biomarker SFI (n = 611)**	0	53.82 (37.83 – 69.82)	0.004**
	1	35.09 (30.61 – 39.57)	
	2	33.87 (31.84 – 35.89)	
	3	34.04 (32.30 – 35.79)	
	4	40.18 (36.27 – 44.09)	
	5	46.96 (34.55 – 59.38)	
**Original 6-biomarker SFI (n = 361)**	0	30.93 (29.54 – 35.32)	0.004**
	1	35.76 (29.75 – 41.76)	
	2	32.61 (30.34 – 34.87)	
	3	32.77 (30.75 – 34.79)	
	4	41.84 (37.32 – 46.36)	
	5	46.34 (31.20 – 61.47)	
	6	n/a	

Abbreviations: SFI, skeletal frailty index; *p*, *p*-value; CI, confidence interval; n, number of cases.
* = $p < 0.1$, ** = $p < 0.05$, *** = $p < 0.001$

Table S2. Cox proportional hazards analysis of original 5- and 6-biomarker SFIs using data on presence vs. absence (binary) and severity/activity (scalar) of individual biomarkers.

**Index**	**Reference Group**	**Index Score**	**Exp(β) (95% CI)**	***p***
**Original 5-biomarker SFI (binary) (n = 611)**	No reference group		0.99 (0.92 – 1.07)	0.88

	Reference: Index score = 0	1	2.11<br>(1.05 – 4.21)	0.04**
		2	2.51<br>(1.28 – 4.92)	0.007**
		3	2.54<br>(1.30 – 4.98)	0.007**
		4	1.74<br>(0.86 – 3.50)	0.12
		5	1.42<br>(0.53 – 3.84)	0.49
**Original 5-biomarker SFI (scalar) (n = 611)**	No reference group		0.89<br>(0.80 – 1.0)	0.044**
**Original 6-biomarker SFI (binary) (n = 361)**	No reference group		0.88<br>(0.80 – 0.98)	0.017**
	Reference: Index score = 0	1	0.67<br>(0.28 – 1.59)	0.36
		2	0.80<br>(0.35 – 1.82)	0.60
		3	0.84<br>(0.37 – 1.91)	0.68
		4	0.43<br>(0.18 – 1.03)	0.06*
		5	0.43<br>(0.13 – 1.42)	0.17
		6	n/a	n/a
**Original 6-biomarker SFI (scalar) (n = 361)**	No reference group		0.82<br>(0.71 - 0.93)	0.003**

Abbreviations: SFI, skeletal frailty index; *p*, *p*-value; CI, confidence interval; n, number of cases.
* = $p < 0.1$, ** = $p < 0.05$, *** = $p < 0.001$

Table S3. Gompertz analysis of original 5- and 6-biomarker SFIs. Results include maximum likelihood estimates of the effect of the index value covariate (with the 95% confidence interval in parentheses) and likelihood ratio tests (LRT) of H_0: Effect of index value covariate = 0 for the original 5- and 6-biomarker SFIs.

Index	Covariate Estimate (95% CI)	LRT	*p*
**Original 5-biomarker SFI (binary)**	9.968<br>(-10.000 − 9.968)	13674528.5	<0.001***

**(n = 611)**			
**Original<br>5-biomarker SFI (scalar)<br>(n = 611)**	10.000<br>(-10.000 − 10.000)	11930718.52	<0.001***
**Original<br>6-biomarker SFI (binary)<br>(n = 361)**	9.598<br>(-10.000 − 9.598)	8131996.3	<0.001***
**Original<br>6-biomarker SFI (scalar)<br>(n = 361)**	9.272<br>(-10.000 − 10.000)	7126035.3	<0.001***

Abbreviations: SFI, skeletal frailty index; *p*, *p*-value; CI, confidence interval; n, number of cases.
* = $p < 0.1$, ** = $p < 0.05$, *** = $p < 0.001$

Table S4. Sample sizes of estimated females and males from medieval cemeteries used in analyses. Sample sizes vary between skeletal frailty (SFI), frailty, and resilience indices, as all adults in these cemeteries could not be observed, measured, or scored for all 4-, 5-, or 6-biomarker criteria.

**Frailty Index**	**East Smithfield**		**Guildhall Yard**		**St. Mary Graces**		**St. Mary Spital**		**Total**
	Female	Male	Female	Male	Female	Male	Female	Male	
5-biomarker skeletal frailty indices (SFI)	29	47	10	5	27	35	249	209	611
6-biomarker skeletal frailty indices (SFI)	13	31	4	4	13	16	148	132	361
5-biomarker frailty indices	19	35	4	2	8	16	140	119	343
4-biomarker frailty indices	35	73	7	6	26	29	217	188	581
4-biomarker resilience indices	35	73	7	6	26	29	217	188	581

Table S5. Skeletal biomarkers of stress incorporated into 5- and 6-biomarker frailty indices (SFI) with scoring schemata and criteria for frailty. Note, observed absence of a condition is considered lowest (“0”) frailty.

**6-biomarker SFI (binary)**				**5-biomarker SFI (binary)**			
**Stress Category**	**Frailty Biomarkers**	**Scoring Observations**	**Frailty Scores**	**Stress Category**	**Frailty Biomarkers**	**Scoring Observations**	**Frailty Scores**
Growth	Linear enamel hypoplasia (LEH)	Presence of mandibular canine with minimally one LEH	1	Growth	Linear enamel hypoplasia (LEH)	Presence of mandibular canine with minimally one LEH	1
	Femoral length	Shortest length (2SD below average OR lowest 25 percentile)	1				
Nutrition and infection	Periosteal new bone formation (PNBF)	Healed, mixed healed/active, active	1	Nutrition and infection	Periosteal new bone formation (PNBF)	Healed, mixed healed/active, active	1
	Periodontal disease	Presence of > 2-mm alveolar resorption	1		Periodontal disease	Presence of > 2-mm alveolar resorption	1
Activity	Osteoarthritis (OA)	Presence of one joint with OA	1	Activity	Osteoarthritis (OA)	Presence of one joint with OA	1
Trauma	Fracture	Antemortem trauma associated with soft tissue injuries or blunt force, sharp force, and projectile	1	Trauma	Fracture	Antemortem trauma associated with soft tissue injuries or blunt force, sharp force, and projectile	1
**6-biomarker SFI (scalar)**				**5-biomarker SFI (scalar)**			
Stress category	Frailty biomarkers	Scoring observations	Frailty scores	Stress category	Frailty biomarkers	Scoring observations	Frailty scores
Growth	Linear enamel hypoplasia (LEH)	1 LEH on canines	0.25	Growth	Linear enamel hypoplasia (LEH)	1 LEH on canines	0.25
		2 LEH on canines	0.5			2 LEH on canines	0.5
		3 LEH on canines	0.75			3 LEH on canines	0.75
		>=4 LEH on canines	1			>=4 LEH on canines	1
	Femoral length	Shortest length (2SD below	1				

		average OR lowest 25 percentile)					
Nutrition and infection	Periosteal new bone formation (PNBF)	Healed	0.33	Nutrition and infection	Periosteal new bone formation (PNBF)	Healed	0.33
		Mixed healed/active	0.66			Mixed healed/active	0.66
		Active	1			Active	1
	Periodontal disease	2-3 mm alveolar resorption	0.33		Periodontal disease	2-3 mm alveolar resorption	0.33
		3-5 mm alveolar resorption	0.66			3-5 mm alveolar resorption	0.66
		>=5 mm alveolar resorption	1			>=5 mm alveolar resorption	1
Activity	Osteoarthritis (OA)	Presence of one joint with OA	1	Activity	Osteoarthritis (OA)	Presence of one joint with OA	1
Trauma	Fracture	Antemortem trauma associated with soft tissue injuries or blunt force, sharp force, and projectile	0.5	Trauma	Fracture	Antemortem trauma associated with soft tissue injuries or blunt force, sharp force, and projectile	0.5
		Antemortem trauma with secondary infection	1			Antemortem trauma with secondary infection	1

Abbreviations: SFI, skeletal frailty index; SD, standard deviation; mm, millimeter

Data S1.

Medieval data (biomarkers, frailty and resilience index values, demographics) for new 5-biomarker frailty indices (binary and scalar) and 4-biomarker frailty and resilience indices (binary and scalar).
